# Prevalence of somatic and psychiatric morbidity across occupations in Switzerland and its correlation with suicide mortality: results from the Swiss National Cohort (1990–2014)

**DOI:** 10.1186/s12888-020-02733-7

**Published:** 2020-06-22

**Authors:** M. Schmid, L. Michaud, N. Bovio, I. Guseva Canu, for the Swiss National Cohort (SNC), Matthias Egger, Adrian Spoerri, Marcel Zwahlen, Milo Puhan, Matthias Bopp, Martin Röösli, Michel Oris, Murielle Bochud

**Affiliations:** 1 Center for Primary Care and Public Health (Unisanté), University of Lausanne, Département Sante, Travail, Environnement (DSTE), Biopôle, Route de la Corniche, 2, 1066 Epalinges-Lausanne, Switzerland; 2 Geneva University Hospital, Geneva, Switzerland; 3 Psychiatric Liaison Service, Lausanne University Hospital, Lausanne, Switzerland

**Keywords:** Suicide, Work-relatedness, Occupational exposure, Psychosocial risk-factors, Concomitant disease, Underreporting

## Abstract

**Background:**

Suicide is a major and complex public health problem. In Switzerland, suicide accounts for about 1000 deaths yearly and is the fourth leading cause of mortality. The first nationwide Swiss study of suicides identified eight male and four female occupations with statistically significant excess of suicide compared to the general Swiss population. Working time, self-employer status, low socio-economic status and low skill level required for occupation were associated with increase in suicide risk. Presently, we aim to compare the distribution of suicide risk across occupations with the prevalence of somatic and psychiatric morbidity in Swiss working-aged adults. We hypothesized that some diseases would cluster in particular occupations, indicating potential work-relatedness of suicides found in these occupations.

**Methods:**

We used the Swiss National Cohort (SNC) and included 10575 males and 2756 females deceased by suicide between 1990 and 2014. We estimated the prevalence of 16 categories of concomitant diseases in each occupation, using national mortality records, and assessed the homogeneity of diseases distribution across occupations. For diseases, which prevalence varied significantly across occupations, we analyzed the correlation with the distribution of suicide risk, estimated as the standardized mortality ratio (SMR) of suicide.

**Results:**

Mental and behavioral disorders were the most commonly reported concomitant diseases in our population. In men, the prevalence of these disorders and more specifically, the prevalence of substance-related and addictive disorders, and of psychotic disorders varied significantly across occupations and was correlated with the SMR of suicide. The prevalence of malignant neoplasms and the prevalence of diseases of the musculoskeletal system and connective tissue also varied significantly across male occupations, while in women, such a variation was observed for neoplasms of uncertain or unknown behavior and diseases of the nervous system and sense organs, without being correlated with the SMR of suicide.

**Conclusion:**

Some of the identified morbidities can be occupation-related and could negatively affect the working capacity and the employability, which in turn could be related to the suicide. Disentangling concomitant diseases according to their work-relatedness and relationship with the suicide risk is important for identifying occupation-related suicides, understanding their characteristics, and developing appropriated interventions for their prevention.

## Background

Suicide is a complex and major health problem worldwide. The estimated annual suicide rate is 10.8 deaths per 100,000 people worldwide; 13,5 deaths per 100′000 male and 7.7 deaths per 100′000 female [1]. In Switzerland, suicide is the fourth leading cause of mortality, accounting for about 1000 deaths per year [2]. Suicide rate varies according to region [3], sex, age [4, 5], calendar period, allowing distinguishing periods of economic crisis [6, 7], geopolitical or military conflict [8] or preventive intervention [9], ethnic origin [10], religion [11], season [12, 13] and occupation [6, 14–16]. The nationwide analysis of suicide mortality across 82 occupations in Switzerland identified eight occupations in men and four occupations in women with statistically significant excess of suicide compared to the general Swiss population [17]. The occupations at risk for both sexes were manufacturing laborers, personal care and related workers and motor vehicle drivers. Moreover, men working as crop and animal producers, agriculture, fishery and related laborers and nursing and midwifery associate professionals, and women working as writers and creative or performing artists had also a significant excess of suicide. This study showed that working time, a self-employer status, low socio-economic status and low skill level required for occupation were risk factors for suicide [17]. However, the study design and shortage of available variables precluded further investigation of occupations at risk of suicide. Such an investigation requests availability of individual and occupational risk factors of suicide for a large sample of workers, which is rarely possible. Most studies conducted in occupations at high suicidal risk, such as agricultural or military workers [18, 19], used medico-administrative data, such as occupation and mortality registries, with no or few individually assessed variables enabling etiological research.

This study aimed to compare the distribution of suicide risk across occupations in Switzerland, with the prevalence of somatic and psychiatric morbidity. Our research hypothesis was that some particular diseases would cluster in particular occupations. The association between the distribution of suicide risk and the prevalence of some of these diseases would thus indicate the possible work-relatedness of the suicide risk in these occupations.

## Methods

### Data sources

Were used the national mortality rates from the Swiss Federal Statistical Office (SFSO) and the Swiss National Cohort (SNC), based on linkage of 1990 and 2000 censuses and mortality records [20, 21]. As the census was mandatory, the SNC covers 98.6% of Swiss population [20]. As the SNC assigned the occupation of the participants, estimation of cause-specific mortality and associated morbidity across Swiss occupations was possible.

### Study sample

We used two study samples. To estimate the risk of suicide by occupation, in the first sample we included all adults from the SNC aged 18–65 years at the moment of the 1990 or 2000 census. We excluded unemployed and job seeking participants and those with unknown occupation to focus on workers. To estimate the prevalence of reported somatic and psychiatric diseases among those deceased by suicide within each occupation, we restricted the second study sample to participants deceased by suicide.

### Definition and coding of outcomes

In Switzerland, the death certificate is mandatory completed by the physician performing the death inspection. This physician (usually an emergency physician, family doctor or forensic physician) should first specify whether the death is due to a natural, violent or undetermined cause. Then, the physician should complete an anonymous form to report the initial, consecutive and concomitant diseases. While the first two diseases correspond to the primary and immediate causes of death, respectively, the concomitant diseases have no causal relationship with death [22]. The SFSO centralizes the completed forms and codes the diseases according to the International Classification of Diseases (ICD) before linkage with the SNC. During 1990–1994, deaths caused by intentional self-harm were coded according to the eighth ICD revision (codes 950–958), and from 1995 onwards according to the tenth ICD revision (codes X60-X84). Suicides were coded as principal cause in all cases except those who died from assisted suicide. In this study, we only considered the former, given that the latter, strictly reserved to specific conditions, are not relevant for investigating the work-related risk factors [23]. For each identified suicide case, two physicians independently examined the reported consecutive and concomitant diseases and assigned them to one of 16 categories of morbidity, according to their ICD code (Table 1).

**Table 1:** International Classification of Diseases (ICD) codes^a^ used for identification and grouping of reported diseases

Type of disease	ICD 8 codes (1990–1994)	ICD 10 codes (1995–2014)
Infectious and parasitic diseases	> = 0 to <=137	^A|^B
Malignant neoplasms	> = 140 to <=209	^C|^D0
Benign neoplasms	> = 210 to <=228	^D1|^D2|^D30|^D31|^D32|^D33|^D34| ^D35|^D36
Neoplasms of uncertain or unknown behaviour	> = 230 to <=339	^D37|^D38|^D39|^D4
Endocrine, nutritional and metabolic diseases	> = 240 to <=279	^E
Diseases of the blood and blood-forming organs	> = 280 to <=289	^D5|^D6|^D7|^D8
Diseases of the nervous system and sense organs	> = 320 to <=389	^G|^H
Diseases of the circulatory system	> = 390 to <=458	^I
Diseases of the respiratory system	> = 460 to <=519	^J
Diseases of the digestive system	> = 520 to <=577	^K
Diseases of the genitourinary system	> = 580 to <=629	^N
Diseases related to pregnancy	(> = 630 to <=678) | (> = 760 to <=779)	^O|^P
Diseases of the skin and subcutaneous tissus	> = 680 to <=709	^L
Diseases of the musculoskeletal system and connective tissue	> = 710 to <=738	^M
Congenital malformations, deformations and chromosomal abnormalitities	> = 740 to <=759	^Q
Mental and behavioural disorders	> = 290 to <=308	^F
Substance-Related and Addictive Disorders	291, 303 and 304	^F1|^F630
Schizophrenia Spectrum and Other Psychotic Disorders	> = 295 and < =299	^F2
Bipolar and Related Disorders/Depressive Disorders	300	^F3
Anxiety Disorders/Obsessive-Compulsive and Related Disorders	307	^F4|^F94|^F631|^F632|^F633|^F634| ^F635|^F636|^F637|^F638|^F639
Personality Disorders/Gender Dysphoria/Disruptive, Impulse-Control and Conduct Disorders/Paraphilic Disorders	301 and 302	^F60|^F61|^F62|^F64|^F65|^F66|^F67| ^F68|^F69|^F91|^F90
Other Psychiatric Disorders	290, 292, 293, 294, 305, 306 and 308	^F0|^F5|^F7|^F8|^F93|^F99

^a^Diseases were coded based on the eighth revision of the International Classification of Diseases from 1990 to 1994 and on the tenth revision from 1995 onwards

^ = which starts with, | = or, >= greater or equal to, <= smaller or equal to

### Definition and coding of occupations

The initial coding of occupations in both censuses was based on the Swiss classification of occupations, subsequently recoded using the four-digit codes of the International Standard Classification of Occupations, version 1988 (ISCO-88) [17]. For this study, we used the two-digit ISCO-88 to aggregate 453 four-digit occupations into 35 occupational groups.

### Statistical analysis

#### Estimation of standardized mortality ratios

Mortality follow-up started either on the 4.12.1990 (1990 census date) or the 5.12.2000 (2000 census date). We followed all participants aged ≥ 18 at the beginning of follow-up up to the earliest date of their 65th birthday, emigration, death or end of the study (31.12.2014). Occupation was treated as time-dependent variable. For each occupational group, person-years were assigned as follows: 1-participants with a single occupation contributed to this occupation for the entire period of their follow-up; 2-participants who changed occupation between 1990 and 2000 census, contributed to the first occupation between 1990 and 2000, and to the second one afterwards till the end of follow-up. To examine the distribution of suicide risk across occupations, we computed standardized mortality ratios (SMRs) as the ratios of every occupation’s observed deaths number to the number of expected deaths. The number of expected deaths was calculated by applying the national cause-specific mortality rates stratified by age and calendar period (both 5-year groups), to the number of person-years for the corresponding calendar period and age group for every occupation. The 95% confidence intervals (IC_95%_) were calculated using the exact Poisson formula [24]. We sorted occupations by increasing SMR value.

#### Estimation of prevalence of reported morbidities

For each occupation, we estimated the prevalence of 16 diseases categories and associated IC_95%_ using the Agresti formula if the sample size within an occupation was ≥40, or using the Jeffreys formula for smaller samples [25, 26].

#### Statistical tests of hypotheses

To test the homogeneity in the prevalence distribution across occupations, we used the Pearson’s Chi2 test of heterogeneity [24], with an alpha-error risk of 5%. We used Holm-Bonferroni [27] correction to account for multiple comparisons. Furthermore, we examined the correlation between the prevalence of diseases and SMR of suicide across occupations for disease, which prevalence varied significantly across occupations. We used Spearman’s correlation coefficient [28] to assess the strength of correlation and assumed an alpha-error risk of 5% when tested its statistical significance. Again, we used Holm-Bonferroni [27] correction to account for multiple comparisons [27]. All analyses were run on STATA, version 15.

## Results

### Description of study sample

The first study sample for estimation of the SMRs consisted of 5,834,618 participants (94,918,456 person-years), 49% of whom were women. The follow-up characteristics were similar in men and women, with mean length of follow-up of 16 years. At study end-point, 4% of participants had died (*n* = 238,504), 19,863 of whom by suicide. Information on occupation was available for 13′331 of them; all were included into the second sample, for estimating the prevalence of reported diseases. This sample comprised 10′575 men and 2′756 women. The mean age at death by suicide was 44.9 ±11.3 in men and 45.2±11.1 in women. Moreover, sex differences were observed in the distribution of participants by occupation, skill-level, and socio-professional category (more unskilled workers and about twice less other self-employed in women). Noteworthy, more than half of women worked 39 h a week or less (52%), compared to only 15% in men (data not shown).

### Risk of suicide

Additional files Table S1 and S2 present the SMR in male and female occupations, respectively. Male agricultural, fishery and related laborers, skilled agricultural and fishery workers, drivers and mobile plant operators experienced statistically significant excess of suicide, compared to general population (Table S1). In stationary plant and related operators, mining, construction, manufacturing and transport laborers, and office clerks, the increase in suicide risk was of borderline significance. In women, the excess of suicide was found among mining, construction, manufacturing and transport laborers and other professionals. Female drivers and mobile plant operators had the highest suicide risk, though of borderline significance (Table S2).

### Prevalence of reported diseases

The prevalence of all reported diseases by occupation (2-digit ISCO-88) in men is presented in Table S1. Table 2 summarizes the prevalence of diseases, for which we observed a statistically significant variation across occupations. The most commonly reported diseases among men were mental and behavioral disorders. The prevalence of malignant neoplasms, diseases of the musculoskeletal system and connective tissue, mental and behavioral disorders and more specifically, substance-related and addictive disorders, schizophrenia spectrum and other psychotic disorders, mood disorders, and anxiety disorders with obsessive-compulsive and related disorders varied significantly across occupations (Table 2).

**Table 2:** Correlation between the age-standardized suicide ratio and the prevalence of concomitant diseases* across occupations among male workers deceased by suicide (SNC, 1990–2014)

Occupation^a^	Nb of suicide	Risk of suicide	Malignant neoplasms	Mental and behavioural disorders	Diseases of the musculoskeletal system and connective tissue	Substance-related and addictive disorders	Schizophrenia Spectrum and other psychotic disorders	Mood disorders	Anxiety, obsessive-compulsive and related disorders
11. Legislators and senior officials	36	0.55 (0.39–0.76)	8.33 (2.40–20.60)	13.89 (5.50–27.80)	0.00 (0.00–6.69)	0.00 (0.00–6.69)	2.78 (0.30–12.26)	11.11 (3.87–24.29)	0.00 (0.00–6.69)
23. Teaching professionals	165	0.65 (0.56–0.76)	4.85 (2.33–9.42)	34.55 (27.71–42.09)	0.00 (0.00–2.74)	3.03 (1.11–7.09)	8.48 (5.02–13.84)	25.45 (19.40–32.63)	0.00 (0.00–2.74)
21. Physical, mathematical and engineering science professionals	497	0.76 (0.69–0.83)	4.23 (2.75–6.41)	31.79 (27.85–36.01)	0.00 (0.00–0.92)	6.04 (4.23–8.51)	5.03 (3.40–7.35)	22.13 (18.70–25.99)	0.00 (0.00–0.92)
12. Corporate managers	775	0.76 (0.71–0.82)	3.74 (2.60–5.34)	26.84 (23.84–30.07)	0.26 (0.01–1.00)	6.19 (4.69–8.13)	3.74 (2.60–5.34)	18.97 (16.36–21.88)	0.00 (0.00–0.59)
31. Physical and engineering science associate professionals	495	0.78 (0.71–0.85)	4.44 (2.92–6.67)	32.32 (28.35–36.57)	0.40 (0.01–1.56)	8.08 (5.97–10.84)	4.44 (2.92–6.67)	21.41 (18.02–25.25)	0.00 (0.00–0.93)
33. Teaching associate professionals	121	0.84 (0.70–1.00)	5.79 (2.63–11.66)	42.98 (34.50–51.88)	0.00 (0.00–3.70)	7.44 (3.79–13.70)	4.13 (1.53–9.56)	30.58 (23.05–39.30)	0.83 (0.00–4.99)
1. Soldiers	12	0.85 (0.44–1.48)	0.00 (0.00–18.53)	41.67 (18.05–68.81)	0.00 (0.00–18.53)	0.00 (0.00–18.53)	0.00 (0.00–18.53)	33.33 (12.45–61.24)	0.00 (0.00–18.53)
13. Managers of small enterprises	158	0.85 (0.72–0.99)	5.06 (2.43–9.83)	27.85 (21.43–35.32)	1.27 (0.05–4.79)	6.96 (3.81–12.16)	1.90 (0.40–5.69)	23.42 (17.46–30.63)	0.00 (0.00–2.86)
24. Other professionals	391	0.86 (0.78–0.95)	6.65 (4.54–9.60)	36.57 (31.95–41.46)	0.26 (0.00–1.58)	9.46 (6.92–12.80)	5.88 (3.91–8.71)	23.79 (19.83–28.26)	0.77 (0.15–2.34)
80. Plant and machine operators and assemblers	31	0.88 (0.62–1.26)	6.45 (1.36–19.12)	38.71 (23.19–56.23)	3.23 (0.35–14.10)	9.68 (2.80–23.63)	0.00 (0.00–7.72)	32.26 (17.94–49.71)	0.00 (0.00–7.72)
91. Sales and services elementary occupations	158	0.89 (0.76–1.04)	1.90 (0.40–5.69)	34.18 (27.23–41.88)	0.63 (0.00–3.86)	7.59 (4.28–12.92)	5.70 (2.88–10.61)	19.62 (14.14–26.54)	3.16 (1.16–7.39)
52. Models, salespersons and demonstrators	219	0.91 (0.80–1.04)	3.20 (1.43–6.58)	29.68 (24.01–36.05)	0.00 (0.00–2.08)	7.76 (4.83–12.15)	2.28 (0.83–5.38)	22.37 (17.34–28.36)	0.46 (0.00–2.80)
10. Legislators, senior officials and managers	90	0.95 (0.77–1.17)	7.78 (3.57–15.44)	31.11 (22.46–41.31)	1.11 (0.00–6.63)	4.44 (1.39–11.23)	0.00 (0.00–4.91)	28.89 (20.50–39.00)	0.00 (0.00–4.91)
22. Life science and health professionals	138	0.95 (0.81–1.13)	4.35 (1.81–9.36)	26.09 (19.45–34.02)	0.00 (0.00–3.26)	4.35 (1.81–9.36)	2.90 (0.88–7.47)	17.39 (11.91–24.63)	1.45 (0.07–5.46)
72. Metal, machinery and related trades workers	944	0.96 (0.90–1.02)	2.33 (1.53–3.52)	36.12 (33.12–39.24)	0.42 (0.12–1.13)	9.22 (7.53–11.24)	6.99 (5.52–8.81)	21.19 (18.70–23.91)	0.95 (0.47–1.83)
71. Extraction and building trades workers	1022	0.96 (0.91–1.02)	1.96 (1.25–3.02)	32.97 (30.16–35.92)	0.78 (0.37–1.57)	11.06 (9.27–13.13)	4.79 (3.63–6.29)	19.86 (17.53–22.42)	0.59 (0.24–1.31)
82. Machine operators and assemblers	262	0.97 (0.86–1.09)	3.05 (1.45–6.01)	37.40 (31.76–43.41)	1.91 (0.69–4.52)	9.92 (6.82–14.19)	5.34 (3.14–8.84)	23.28 (18.56–28.78)	1.15 (0.23–3.47)
34. Other associate professionals	1012	1.00 (0.94–1.07)	3.36 (2.40–4.67)	30.24 (27.49–33.14)	0.59 (0.24–1.32)	6.42 (5.06–8.11)	4.05 (2.99–5.46)	21.25 (18.83–23.87)	0.40 (0.11–1.05)
74. Other craft and related trades workers	380	1.01 (0.91–1.11)	1.84 (0.82–3.83)	32.11 (27.61–36.96)	1.58 (0.64–3.49)	11.58 (8.72–15.21)	4.74 (2.97–7.41)	18.16 (14.59–22.36)	0.53 (0.02–2.03)
42. Customer services clerks	136	1.02 (0.86–1.20)	2.21 (0.46–6.57)	37.50 (29.80–45.88)	0.00 (0.00–3.30)	6.62 (3.36–12.26)	7.35 (3.90–13.15)	25.74 (19.10–33.71)	0.00 (0.00–3.30)
51. Personal and protective services workers	544	1.02 (0.94–1.11)	3.13 (1.92–4.98)	34.01 (30.15–38.09)	0.74 (0.21–1.95)	9.19 (7.02–11.93)	5.88 (4.17–8.21)	20.77 (17.57–24.39)	0.37 (0.01–1.42)
73. Precision, handicraft, craft printing & related trade workers	150	1.03 (0.88–1.21)	2.67 (0.81–6.89)	36.67 (29.37–44.63)	0.00 (0.00–3.00)	6.67 (3.52–11.97)	3.33 (1.22–7.77)	26.00 (19.62–33.58)	1.33 (0.06–5.04)
93. Labourers in mining, construction, manufacturing & transport	860	1.06 (0.99–1.13)	1.51 (0.86–2.60)	32.21 (29.17–35.41)	0.23 (0.01–0.90)	11.40 (9.43–13.70)	9.30 (7.53–11.44)	13.72 (11.58–16.19)	0.35 (0.07–1.07)
41. Office clerks	677	1.08 (1.00–1.17)	2.36 (1.43–3.83)	32.05 (28.65–35.66)	0.59 (0.17–1.57)	8.71 (6.80–11.09)	6.06 (4.48–8.13)	19.05 (16.27–22.19)	0.44 (0.09–1.36)
83. Drivers and mobile plant operators	514	1.11 (1.02–1.21)	1.75 (0.87–3.35)	32.68 (28.77–36.86)	0.58 (0.11–1.79)	9.34 (7.10–12.18)	4.86 (3.29–7.11)	20.04 (16.80–23.72)	0.19 (0.00–1.21)
32. Life science and health associate professionals	108	1.12 (0.93–1.36)	4.63 (1.72–10.65)	37.04 (28.51–46.45)	0.00 (0.00–4.13)	12.04 (7.04–19.64)	3.70 (1.14–9.44)	24.07 (16.94–32.99)	0.00 (0.00–4.13)
61. Skilled agricultural and fishery workers	575	1.13 (1.04–1.22)	1.91 (1.03–3.44)	40.70 (36.75–44.76)	2.09 (1.16–3.65)	6.96 (5.13–9.35)	6.61 (4.83–8.96)	28.17 (24.65–31.99)	0.52 (0.10–1.60)
81. Stationary plant and related operators	59	1.23 (0.96–1.59)	1.69 (0.00–9.85)	42.37 (30.60–55.07)	0.00 (0.00–7.31)	8.47 (3.27–18.75)	6.78 (2.20–16.64)	25.42 (15.96–37.89)	0.00 (0.00–7.31)
92. Agricultural, fishery and related labourers	35	1.42 (1.02–1.98)	0.00 (0.00–6.88)	48.57 (32.68–64.69)	0.00 (0.00–6.88)	22.86 (11.44–38.55)	8.57 (2.47–21.14)	25.71 (13.55–41.71)	0.00 (0.00–6.88)
Spearman’s correlation with Holm-Bonferroni correction (*p*-value < 0.01**, < 0.05*)			− 0.54*	0.36	0.10	0.63**	0.51*	−0.07	0.16

*Only diseases, which prevalence varied statistically significantly across occupations are presented. For each disease, the prevalence is reported with associated 95%-confidence interval (95%-CI)

^a^Occupations are coded based on the 2-digit International Standard Classification of Occupations, version 88 and limited to those with at least 10 observed suicides; Occupations are sorted by increasing risk of suicide, reported as the age-standardized suicide ratio (SMR) and associated 95%-CI

Malignant neoplasms were highly prevalent among legislators and senior officials, legislators, senior officials and managers not otherwise specified and among other professionals (ISCO code 24, Table 2). The lowest prevalence of these diseases was observed among soldiers, clerks, craft and related trades workers, and agricultural, fishery and related laborers. Diseases of the musculoskeletal system and connective tissue were more prevalent among plant machine operators, skilled agricultural and fishery workers, and machine operators and assemblers, than in all other occupations. Agricultural, fishery and related laborers, teaching associate professionals and stationary plant and related operators had the highest prevalence of mental and behavioral disorders, while the legislators and senior officials, craft and related trades workers, and life science and health professionals had the lowest prevalence. Among four subcategories of psychiatric diseases, mood disorders were the most reported. They were highly prevalent among soldiers, plant and machine operators and assemblers, and teaching associate professionals, while clerks along with legislators and senior officials had the lowest prevalence. Substance-related and addictive disorders were highly prevalent among agricultural, fishery, and related laborers, clerks, and life science and health associate professionals. Soldiers, craft and related trades workers, and legislators and senior officials had the lowest reported prevalence of these diseases. Schizophrenia spectrum and other psychotic disorders were prevalent among clerks, laborers in mining, construction, manufacturing and transport, and agricultural, fishery and related laborers. Soldiers, legislators, senior officials and managers, craft and related workers along with plant and machine operators and assemblers were almost exempted of them, according to the reported data. The reporting of anxiety disorders/obsessive-compulsive and related disorders was rarer than reporting of other subgroups of psychiatric diseases. Sales and services elementary occupations, life science and health associate professionals, precision, handicraft, craft printing and related trades workers, and machine operators and assemblers had the highest prevalence of anxiety and related disorders.

Table S2 presents the prevalence of all reported diseases by occupations in women, while Table 3 presents the prevalence of diseases with statistically significant variation across occupations. Mental and behavioral disorders were the most commonly reported diseases. Among all considered diseases, only two groups presented statistically significant variation of prevalence across occupations, the neoplasms of uncertain or unknown behavior and the diseases of the nervous system and sense organs (Table 3). The former was rarely reported; essentially in legislators, senior officials and managers and other professionals. Diseases of the nervous system and sense organs were the most prevalent among metal, machinery and related trades workers, precision, handicraft, craft printing and related workers, and among legislators, senior officials, and managers. The lowest prevalence was observed among drivers and mobile plant operators and among physical, mathematical and engineering science professionals (Table 3).

**Table 3:** Correlation between the age-standardized suicide ratio and the prevalence of concomitant diseases* across occupations among female workers deceased by suicide (SNC, 1990–2014)

Occupationa	Nb of suicide	Risk of suicide	Neoplasms of uncertain or unknown behaviour	Diseases of the nervous system and sense organs
74. Other craft and related trades workers	29	0.65 (0.44–0.93)	0.00 (0.00–8.23)	3.45 (0.37–15.01)
61. Skilled agricultural and fishery workers	46	0.65 (0.49–0.87)	0.00 (0.00–9.20)	2.17 (0.00–12.38)
13. Managers of small enterprises	23	0.68 (0.43–1.02)	0.00 (0.00–10.24)	4.35 (0.47–18.58)
72. Metal, machinery and related trades workers	12	0.69 (0.35–1.20)	0.00 (0.00–18.53)	8.33 (0.91–32.85)
23. Teaching professionals	78	0.78 (0.63–0.98)	0.00 (0.00–5.63)	2.56 (0.16–9.42)
12. Corporate managers	87	0.84 (0.68–1.04)	0.00 (0.00–5.07)	2.30 (0.14–8.49)
91. Sales and services elementary occupations	113	0.84 (0.69–1.00)	0.00 (0.00–3.95)	3.54 (1.09–9.04)
52. Models, salespersons and demonstrators	216	0.87 (0.76–0.99)	0.00 (0.00–2.10)	4.17 (2.10–7.84)
33. Teaching associate professionals	108	0.88 (0.73–1.06)	0.00 (0.00–4.13)	3.70 (1.14–9.44)
31. Physical and engineering science associate professionals	55	0.93 (0.71–1.21)	0.00 (0.00–7.80)	5.45 (1.29–15.44)
41. Office clerks	515	0.93 (0.85–1.01)	0.00 (0.00–0.89)	2.14 (1.15–3.83)
82. Machine operators and assemblers	31	0.97 (0.68–1.38)	0.00 (0.00–7.72)	3.23 (0.35–14.10)
10. Legislators, senior officials and managers	16	0.99 (0.57–1.62)	6.25 (0.68–25.69)	6.25 (0.68–25.69)
32. Life science and health associate professionals	232	1.02 (0.90–1.16)	0.00 (0.00–1.96)	2.16 (0.78–5.09)
51. Personal and protective services workers	349	1.03 (0.93–1.15)	0.00 (0.00–1.31)	2.01 (0.89–4.17)
34. Other associate professionals	230	1.04 (0.91–1.18)	0.00 (0.00–1.98)	3.04 (1.36–6.27)
21. Physical, mathematical and engineering science professionals	24	1.05 (0.67–1.57)	0.00 (0.00–9.84)	0.00 (0.00–9.84)
42. Customer services clerks	103	1.10 (0.91–1.33)	0.00 (0.00–4.32)	2.91 (0.63–8.58)
73. Precision, handicraft, craft printing and related trade workers	31	1.15 (0.81–1.63)	0.00 (0.00–7.72)	6.45 (1.36–19.12)
93. Labourers in mining, construction, manufacturing and transport	263	1.19 (1.05–1.34)	0.00 (0.00–1.73)	1.52 (0.45–3.99)
22. Life science and health professionals	31	1.22 (0.86–1.74)	0.00 (0.00–7.72)	3.23 (0.35–14.10)
24. Other professionals	133	1.22 (1.03–1.44)	0.75 (0.00–4.56)	4.51 (1.88–9.70)
83. Drivers and mobile plant operators	13	1.83 (0.98–3.14)	0.00 (0.00–17.26)	0.00 (0.00–17.26)
Spearman’s [5] correlation with Holm-Bonferroni correction (*p*-value < 0.01**, < 0.05*)			0.20	−0.05

*Only diseases, which prevalence varied statistically significantly across occupations are presented. For each disease, the prevalence is reported with associated 95%-confidence interval (95%-CI)

^a^Occupations are coded based on the 2-digit International Standard Classification of Occupations, version 88 and limited to those with at least 10 observed suicides; Occupations are sorted by increasing risk of suicide, reported as the age-standardized mortality ratio (SMR) and associated 95%-CI

### Correlation between the prevalence of reported diseases and the risk of suicide

In men, the prevalence of malignant neoplasms was negatively correlated with the SMRs (Table 2). In contrast, the prevalence of substance related and addictive disorders and schizophrenia spectrum and other psychotic disorders, was positively correlated with the SMRs. In women, we found no significant correlation (Table 3).

## Discussion

Mental and behavioral disorders were the most commonly reported diseases in men and women deceased by suicide. In men, the prevalence of these disorders along with the prevalence of malignant neoplasms, diseases of the musculoskeletal system and connective tissue varied significantly across occupational groups. In women, such a variation was observed only for neoplasms of uncertain or unknown behavior and diseases of the nervous system and sense organs. The prevalence of these diseases was not correlated with the risk of suicide in women, while in men, we found two positive statistically significant correlations, for substance related and addictive disorders and for schizophrenia spectrum and other psychotic disorders.

From the causal inference perspective, the temporality in the relation between these diseases and occupations with the highest prevalence plays a dual but crucial role. On the one hand, the presence or the onset of a disease can influence the choice of one’s occupation, for instance, when a physical disability precludes the performance of certain tasks or when the development of psychiatric symptoms before the end of occupational training hampers the recruitment in some occupations. On the other hand, an occupation can cause or exacerbate a disease, such as anxiety disorders, or substance use disorders. In both cases, some diseases could be causally related with suicide risk, but only a part of them could be occupation-related. This part of diseases is particularly interesting form the suicide preventive perspective, as their primary prevention at workplaces would subsequently reduce the risk of suicide. The other part of diseases, related with suicidal risk, clusters in particular occupations due to socio-demographic, cultural or medical selection mechanisms, without being occupation-related. In this case, the secondary and tertiary prevention of such diseases could also prevent suicides if implemented at workplaces, to facilitate early detection and encourage their treatment.

Moreover, assessing the extent to which occupational diseases may influence suicide allows identifying occupational groups at risk deserving appropriate prevention. Therefore, each disease needs to be analyzed separately.

### Psychiatric morbidity

#### Substance related and addictive disorders

Substance related and addictive disorders, for which harmful use of alcohol was the most common disorder, were positively associated with the SMR in men but not in women. Furthermore, their prevalence in men was higher in agricultural, fishery and related laborer, and clerks and lower in soldiers, legislators and senior officials, and craft and related trades workers. Alcoholism is closely associated with suicide [29] and varies across occupations. Heavy drinkers choose occupations where there is tolerance towards alcohol misuse and where drinking habits are sharing by colleagues [30]. For instance, in Finland, men working in construction, craft work services and some elementary occupations have the highest risk of alcoholism [31]. In Sweden, 21 male and 5 female occupations, mostly manual, had a twice-higher risk of alcoholism compared to the general population [32]. The alcoholism in these occupations is likely to be occupation-related, either as a reaction against the work stress (in drivers, after a work-shift) or as a work habit (in managers during business diners and events) [33]. This also applies to elementary occupations in both sexes, where the lack of reward and low decisional attitude are the known psychosocial risk-factors [34, 35] and where we found the highest risk of suicide in Switzerland [17]. As the alcoholism in these occupations seems work-related, specific prevention programs should be designed for these occupations in order to reduce the associated suicide risk.

#### Schizophrenia spectrum and other psychotic disorders

Schizophrenia was the main diagnosis within this disorder category. We found no studies on prevalence of psychotic disorders across occupations. Palmer et al. estimated the lifetime risk of suicide at 4.9% of schizophrenics and reported that being young, male, unemployed and having a higher level of education as risk-factors of schizophrenia [36]. We found a relatively low prevalence of schizophrenia in occupations with high level of education. As the symptoms of schizophrenia usually appear during early adulthood, it could impede a high education achievement and recruitment as professional. This may explain why we found high prevalence of schizophrenia in elementary occupations. Schizophrenia and other psychotic disorders were positively correlated with suicide risk. Nevertheless, it seems that diagnosis of schizophrenia and other psychotic disorders is not induced by occupation but rather predetermines the choice of occupation. Therefore, the occupations with high prevalence of these disorders could be targeted for their secondary prevention to prevent suicide.

#### Mood disorders

Mood disorder was the most prevalent category of diseases observed in our study. Most were depressive disorders. Soldiers, teaching associates professionals, legislators, senior officials, and managers had the highest prevalence of mood disorders. Many studies documented the relationship between occupation, depression and suicide, especially among health professionals [37–41], but none described the prevalence of mood disorders across occupations. In this study, the number of soldiers was too low (n=12) for a meaningful interpretation. Among teachers, the workload, especially a high number of students in classrooms, low self-esteem, and lack of satisfaction in their occupation are occupational risk factors of depression/burnout [42–45]. Swiss teachers were found at high risk of work-stress and resulting depression [46], but not at risk of suicide [17]. In contrast, Swiss female managers appear at risk of both mood disorder and suicides [17, 47]. Further research should document their occupational exposure and their relationship with mood disorders and suicide to address their work-relatedness.

#### Anxiety disorders/obsessive-compulsive and related disorders

Sales and services elementary occupations, life science and health professionals, and precision, handicraft, craft printing and related trades workers had a relatively high prevalence of these disorders. A meta-analysis revealed that people with anxiety were at risk of developing suicide ideation, behaviors and attempts [48]. Furthermore, generalized social anxiety and major depression seem associated with the same risk of suicide attempts [49]. A high prevalence of anxiety (48%) was recently confirmed in mental health nurses [50]. Healthcare professionals in general face the stressful situations as exposure to pain, deaths, excessive workloads and lack of supports [51, 52], which could contribute to anxiety disorder development. The same applies to sales and services elementary occupations, where job insecurity and stressful interpersonal relations (high demands of clients and managers) were associated with increased anxiety and development of suicide ideation [53, 54]. Work conditions play an important role in development of anxiety. High job demand, low job control, role stress, lack of rewards, job insecurity and working hours are reported as occupational risk factors of anxiety [55–57]. Many of them are also risk factors of depression. Given the documented relationships between depression and suicide and anxiety and suicide, primary prevention seems suitable in occupations with a high prevalence of these disorders.

### Somatic morbidities

#### Neoplasms

We found a significant occupational variation in the prevalence of malignant neoplasms in men and of neoplasms of unknown or uncertain behavior in women. For the latter, results are difficult to interpret given the heterogeneity of neoplasms in this category and the low number of cases. Moreover, caution is needed regarding our finding on the negative correlation between the prevalence of malignant neoplasms and the suicide risk in men, as it contradicts all available scientific evidence. Indeed, prior studies showed that the risk of suicide increased significantly in the first year after a diagnosis of malignant neoplasm, and varied depending on the cancer type [58, 59]. Lack of support and referral to psychiatrist was often reported in studies of cancer patients, suggesting a negative effect of this disease on mental health [58, 60]. Moreover, it is unlikely that officials, and managers not otherwise specified, which we identified with the highest prevalence of malignant neoplasms, were exposed to occupational carcinogens. Although we think the negative correlation between cancer and suicide unlikely, further analyses are needed.

#### Diseases of the musculoskeletal system and connective tissue

This category encompasses different diseases, having a common fact, pain. Pain, especially when it becomes chronic, is a known risk factor of suicide [61]. There is an increase in the recognition of work-related musculoskeletal disease, such as neck pain [62, 63], back pain [64, 65], or autoimmune connective tissue disease [66], although these are less recognized in Switzerland [67]. In our study, diseases of the musculoskeletal system and connective tissue were mostly prevalent among plant machine operators, skilled agricultural and fishery workers and machine operators and assemblers. Working in these physically demanding occupations could be difficult once the pain became chronic. However, changing or leaving such an occupation at an advanced age and without any financial compensation may have a negative impact on both professional and social life. This could have an influence on the suicidal process. Although the prevalence of these diseases was generally low, primary pain prevention through ergonomic and organizational improvements in physically demanding and sedentary workplaces could have a positive impact on the prevention of work-related suicide.

#### Diseases of the nervous system and sense organs

Despite a low prevalence of these diseases in our study, we found a statistically significant variation across female occupations. The most frequent diseases were multiple sclerosis and amyotrophic lateral sclerosis, both diagnosed at the younger age compared to other neurological diseases (e.g. Alzheimer, Huntington, and Parkinson diseases) [68]. Many neurological diseases, including amyotrophic lateral sclerosis, multiple sclerosis, and epilepsy, have been linked to suicide [69–72]. In patients with multiple sclerosis, the main risk factors of suicide are depression, its severity, social isolation, and alcohol abuse [70]. Neurological and psychiatric diseases can cause each other and be caused by the same factors. For example, brain lesions from stroke or multiple sclerosis could induce psychiatric diseases [72, 73]. Moreover, in some cases, a psychiatric disorder may announce the onset of a neurological disorder [72]. The relationship between neurological disorders and occupation is disease-dependent. Some occupational exposures, including heavy physical work, professional sports, metals, chemicals, electromagnetic fields, or working with electricity and in healthcare were significantly related with risk of amyotrophic lateral sclerosis [74]. For multiple sclerosis, occupational risk factors comprise shift-work, exposure to solvents, radiation, and outdoor work in agriculture [75–77]. In our study, metal, machinery and related trades worker; precision, handicraft, craft printing and related workers; and legislators, senior officials and managers had the highest prevalence of diseases of the nervous system and sense organs. However, the low number of cases made it difficult to interpret. Nevertheless, it is likely that some occupational risk-factors contributed to the development of neurological diseases in these occupations (e.g., heavy physical work, shift work in the first two occupations) and that suicide was related to neurological disease (e.g., as consequence loss of autonomy). It is also possible that the development of a neurological disease in these occupations has greater consequences on employability, and therefore contributes to a higher suicidality. Further research should verify these hypotheses and suggest appropriate recommendations.

### Strengths and limits of study

This study is one of the few to analyze suicide on a national scale. The inclusion of women in our analysis is one of the main strengths. Indeed, they have been frequently excluded from studies of occupation-related suicide, as suicide is a rare event in female population of working age. The current ratio of male to female suicides, characterizing the magnitude of gender gap in national age-standardized suicide mortality [78] is 1.75 in Switzerland. However, the number of female suicides deaths included in this study is 3.8-times smaller compared to males, as this study was restricted to working-age participants with known occupational activity. Therefore, this difference is likely to reflect the gender inequalities in suicide mortality, operated through the occupation access restrictions of Swiss women because of motherhood and/or family duties [79, 80] in addition to the already described educational inequalities in suicide [81].

Given the absence of individual data on morbidity in Switzerland, as in most countries, addressing the work-relatedness of suicides and identifying their occupational risk-factors remain difficult. An investigation of the temporality between the onset or diagnosis of the disease and the start and end dates of occupation requires individual data that were unavailable in the SNC. The use of Spearman’s correlation between the SMR of suicide and prevalence of concomitant diseases is less robust than would be a multivariate regression, accounting for potential confounders and interactions between variables. Therefore, from the causal inference perspective, such an analysis is limited due to its inability to conform causality and rule out bi-directional relationships and confounding factors. With this respect, individual assessment of work-related stress and exposure to other risk factors of suicide in the occupations at risk of suicide identified in this study will be necessary.

The use of national mortality statistics allowed us to assess the prevalence of concomitant diseases homogenously, though regardless of the above-mentioned temporality. There is concern, however, of an underreporting of concomitant diseases. Indeed, psychological autopsy identifies psychiatric disorders in about 90% of suicide cases [82]. The low prevalence of psychiatric disorders in our study reflects therefore the magnitude of this underreporting. Because the disease reporting is independent on occupations, this underreporting is not a bias. However, it decreases the precision in prevalence estimates, reducing the statistical power of our study.

The reasons of underreporting are multiple and represent potential targets for improvement. They comprise 1-an insufficient education during medical curriculum on how to report diseases; 2-an insufficient physicians’ attention on the importance of an accurate reporting of concomitant diseases for the accuracy of national health statistics and therefore, for the public health and medical research activities; 3-an insufficient valorization of the reporting activities, which are perceived as time-consuming, financially inefficient, and increasing the load of administrative work concurrently with clinical activity. However, the most important reason is the lack of information on existing morbidities that the death-inspecting physician can properly report. The diagnosis of psychiatric disorders are usually underdiagnosed prior to death. However, even when diagnosis was made, it often remains unknown to the death-inspecting physician. Depending on the context and place of death, information on comorbidity can be provided by the relatives of the deceased person, by a shared hospital records, or by the person’s general practitioner. Although the first source of information is the least precise, it is one of the easiest available in practice. The use of shared electronic medical records is still limited in Switzerland, and some general practitioners still use paper medical records. As a remediating strategy, one could consider to invest these last two issues and stimulate a safely record sharing.

While awaiting for these improvements, available disease registries can be used as alternative source of morbidity data. In Switzerland, the cancer registry is now mandatory in all cantons, enabling a cross-linkage between our cohort and cancer registry data. This possibility is of particular interest, as the prevalence of neoplasms was unequally distributed across occupations in both men and women. Similarly, to better assess the prevalence of neurological diseases, which prevalence varied across occupations in women and can be occupation-related, a registry of such diseases would be of great interest. The registries of psychiatric diseases may raise discrimination-related concerns, and their benefit from the public health and prevention perspective is questionable. Therefore, other sources of accurate information should be sought. In meantime, strategies improving the quality of diseases reporting in the SFSO forms should be developed. In this respect, the mandatory French reporting on the death certificate of the place where the suicide was committed and the potential work-related reasons for the suicide could be an example to follow [83].

## Conclusion

Mental and behavioral disorders were the most commonly reported concomitant diseases in men and women deceased by suicide between 1990 and 2014 in Switzerland. In men, the prevalence of these disorders and more specifically, substance-related and addictive disorders, and schizophrenia spectrum disorders varied significantly across occupations and was correlated with the age-standardized risk of suicide. The prevalence of malignant neoplasms and diseases of the musculoskeletal system and connective tissue also varied significantly across male occupations, while in women, such a variation was observed for neoplasms of uncertain or unknown behavior and diseases of the nervous system and sense organs, without being correlated with the risk of suicide. These findings are original and should be further confirmed. Nevertheless, they open new preventive possibilities and encourage seeing suicide not only as related to psychiatric illnesses but to a broader societal and, in particular, occupational environment, which is auspicious for carrying out targeted preventive actions.

## Supplementary information

**Additional file 1 Table S1.** Pearson’s Chi2 test of heterogeneity in prevalence of reported diseases with associated 95% confidence interval across occupations^a^ among male workers deceased by suicide (SNC, 1990–2014)

**Additional file 2 Table S2.** Pearson’s Chi2 test of heterogeneity in prevalence of reported diseases with associated 95% confidence interval across occupational groups^a^ among female workers deceased by suicide (SNC, 1990–2014)

## Data Availability

Individual data from different data sets were used for the construction of the SNC. All these data are the property of the Swiss Federal Statistical Office (SFSO) and can only be made available by legal agreements with the SFSO. This also applies to derivatives such as the analysis files used for this study. However, after approval of the SNC Scientific Board, a specific SNC module contract with SFSO would allow researchers to receive analysis files for replication of the analysis. Data requests should be sent to Prof. Milo Puhan (chairman of the SNC Scientific Board, miloalan.puhan@uzh.ch).

## Abbreviations

SNC: Swiss National Cohort
SMR: Standardized Mortality Ratio
SFSO: Swiss Federal Statistical Office
ICD: International Classification of Diseases
ISCO-88: International Standard Classification of Occupations, version 88
NSP: *Nomenclature Suisse des Professions*
