# Supplementary material for: Prevalence of somatic and psychiatric morbidity across occupations in Switzerland and its correlation with suicide mortality: results from the Swiss National Cohort (1990–2014)
Source: BMC Psychiatry. 2020 Jun 22;20:324. doi: 10.1186/s12888-020-02733-7 (PMC7310107; doi:10.1186/s12888-020-02733-7)
Supplement: Supplementary file 1 — Additional file 1 Table S1. Pearson’s Chi2 test of heterogeneity in prevalence of reported diseases with associated 95% confidence interval across occupationsa among male workers deceased by suicide (SNC, 1990–2014) [file 12888_2020_2733_MOESM1_ESM.docx]

| **Table S1.** Pearson’s Chi2 test of heterogeneity in prevalence of reported diseases with associated 95% confidence interval across occupations^a^ among male workers deceased by suicide (SNC, 1990-2014) | | | | | | | | | | |  | |  |  |
| --- | --- | --- | --- | --- | --- | --- | --- | --- | --- | --- | --- | --- | --- | --- |
| Occupation 2-digit ISCO (88) | Nb of suicide^b^ | SMR^c^ | Infectious and  parasitic diseases | Malignant neoplasms | Benign neoplasms | Neoplasms of uncertain or unknown behaviour | Diseases of the blood and blood-forming organs | Mental and behavioural disorders | Diseases of the nervous system and sense organs | Diseases of the circulatory system | |  |  |  |
| 11. Legislators and senior officials | 36 | 0.55 (0.39-0.76) | 0.00 (0.00-6.69) | 8.33 (2.40-20.60) | 0.00 (0.00-6.69) | 0.00 (0.00-6.69) | 0.00 (0.00-6.69) | 13.89 (5.50-27.80) | 2.78 (0.30-12.26) | 2.78 (0.30-12.26) | |  |  |  |
| 23. Teaching professionals | 165 | 0.65 (0.56-0.76) | 3.03 (1.11-7.09) | 4.85 (2.33-9.42) | 0.00 (0.00-2.74) | 0.00 (0.00-2.74) | 0.00 (0.00-2.74) | 34.55 (27.71-42.09) | 3.64 (1.50-7.88) | 3.64 (1.50-7.88) | |  |  |  |
| 21. Physical, mathematical and engineering science professionals | 497 | 0.76 (0.69-0.83) | 1.41 (0.62-2.94) | 4.23 (2.75-6.41) | 0.00 (0.00-0.92) | 0.00 (0.00-0.92) | 0.20 (0.00-1.25) | 31.79 (27.85-36.01) | 2.62 (1.49-4.47) | 2.01 (1.05-3.72) | |  |  |  |
| 12. Corporate managers | 775 | 0.76 (0.71-0.82) | 0.65 (0.23-1.55) | 3.74 (2.60-5.34) | 0.00 (0.00-0.59) | 0.00 (0.00-0.59) | 0.00 (0.00-0.59) | 26.84 (23.84-30.07) | 0.90 (0.40-1.89) | 1.42 (0.76-2.56) | |  |  |  |
| 31. Physical and engineering science associate professionals | 495 | 0.78 (0.71-0.85) | 0.81 (0.24-2.14) | 4.44 (2.92-6.67) | 0.00 (0.00-0.93) | 0.00 (0.00-0.93) | 0.00 (0.00-0.93) | 32.32 (28.35-36.57) | 2.63 (1.50-4.49) | 1.82 (0.91-3.47) | |  |  |  |
| 33. Teaching associate professionals | 121 | 0.84 (0.70-1.00) | 2.48 (0.53-7.35) | 5.79 (2.63-11.66) | 0.00 (0.00-3.70) | 0.00 (0.00-3.70) | 0.00 (0.00-3.70) | 42.98 (34.50-51.88) | 2.48 (0.53-7.35) | 0.00 (0.00-3.70) | |  |  |  |
| 1. Soldiers | 12 | 0.85 (0.44-1.48) | 0.00 (0.00-18.53) | 0.00 (0.00-18.53) | 0.00 (0.00-18.53) | 0.00 (0.00-18.53) | 0.00 (0.00-18.53) | 41.67 (18.05-68.81) | 0.00 (0.00-18.53) | 0.00 (0.00-18.53) | |  |  |  |
| 13. Managers of small enterprises | 158 | 0.85 (0.72-0.99) | 0.00 (0.00-2.86) | 5.06 (2.43-9.83) | 0.63 (0.00-3.86) | 0.00 (0.00-2.86) | 0.00 (0.00-2.86) | 27.85 (21.43-35.32) | 1.90 (0.40-5.69) | 1.90 (0.40-5.69) | |  |  |  |
| 24. Other professionals | 391 | 0.86 (0.78-0.95) | 1.28 (0.46-3.05) | 6.65 (4.54-9.60) | 0.00 (0.00-1.17) | 0.00 (0.00-1.17) | 0.00 (0.00-1.17) | 36.57 (31.95-41.46) | 1.02 (0.30-2.70) | 2.05 (0.97-4.06) | |  |  |  |
| 80. Plant and machine operators and assemblers | 31 | 0.88 (0.62-1.26) | 0.00 (0.00-7.72) | 6.45 (1.36-19.12) | 0.00 (0.00-7.72) | 0.00 (0.00-7.72) | 0.00 (0.00-7.72) | 38.71 (23.19-56.23) | 3.23 (0.35-14.10) | 3.23 (0.35-14.10) | |  |  |  |
| 91. Sales and services elementary occupations | 158 | 0.89 (0.76-1.04) | 0.00 (0.00-2.86) | 1.90 (0.40-5.69) | 0.00 (0.00-2.86) | 0.00 (0.00-2.86) | 0.00 (0.00-2.86) | 34.18 (27.23-41.88) | 1.27 (0.05-4.79) | 2.53 (0.77-6.55) | |  |  |  |
| 52. Models, salespersons and demonstrators | 219 | 0.91 (0.80-1.04) | 0.91 (0.03-3.49) | 3.20 (1.43-6.58) | 0.00 (0.00-2.08) | 0.00 (0.00-2.08) | 0.00 (0.00-2.08) | 29.68 (24.01-36.05) | 2.28 (0.83-5.38) | 1.37 (0.28-4.14) | |  |  |  |
| 10. Legislators, senior officials and managers | 90 | 0.95 (0.77-1.17) | 0.00 (0.00-4.91) | 7.78 (3.57-15.44) | 0.00 (0.00-4.91) | 1.11 (0.00-6.63) | 0.00 (0.00-4.91) | 31.11 (22.46-41.31) | 2.22 (0.13-8.23) | 4.44 (1.39-11.23) | |  |  |  |
| 22. Life science and health professionals | 138 | 0.95 (0.81-1.13) | 0.72 (0.00-4.40) | 4.35 (1.81-9.36) | 0.72 (0.00-4.40) | 0.00 (0.00-3.26) | 0.72 (0.00-4.40) | 26.09 (19.45-34.02) | 0.00 (0.00-3.26) | 1.45 (0.07-5.46) | |  |  |  |
| 72. Metal, machinery and related trades workers | 944 | 0.96 (0.90-1.02) | 0.42 (0.12-1.13) | 2.33 (1.53-3.52) | 0.11 (0.00-0.66) | 0.00 (0.00-0.49) | 0.00 (0.00-0.49) | 36.12 (33.12-39.24) | 1.27 (0.70-2.23) | 1.59 (0.94-2.63) | |  |  |  |
| 71. Extraction and building trades workers | 1022 | 0.96 (0.91-1.02) | 0.68 (0.30-1.44) | 1.96 (1.25-3.02) | 0.10 (0.00-0.61) | 0.00 (0.00-0.45) | 0.10 (0.00-0.61) | 32.97 (30.16-35.92) | 1.47 (0.87-2.43) | 1.76 (1.10-2.79) | |  |  |  |
| 82. Machine operators and assemblers | 262 | 0.97 (0.86-1.09) | 0.38 (0.00-2.35) | 3.05 (1.45-6.01) | 0.00 (0.00-1.74) | 0.00 (0.00-1.74) | 0.00 (0.00-1.74) | 37.40 (31.76-43.41) | 1.91 (0.69-4.52) | 2.67 (1.19-5.52) | |  |  |  |
| 34. Other associate professionals | 1012 | 1.00 (0.94-1.07) | 0.79 (0.37-1.58) | 3.36 (2.40-4.67) | 0.00 (0.00-0.46) | 0.10 (0.00-0.62) | 0.00 (0.00-0.46) | 30.24 (27.49-33.14) | 1.78 (1.11-2.81) | 1.38 (0.80-2.33) | |  |  |  |
| 74. Other craft and related trades workers | 380 | 1.01 (0.91-1.11) | 1.05 (0.31-2.78) | 1.84 (0.82-3.83) | 0.00 (0.00-1.21) | 0.00 (0.00-1.21) | 0.00 (0.00-1.21) | 32.11 (27.61-36.96) | 1.32 (0.47-3.13) | 0.26 (0.00-1.63) | |  |  |  |
| 42. Customer services clerks | 136 | 1.02 (0.86-1.20) | 2.21 (0.46-6.57) | 2.21 (0.46-6.57) | 0.00 (0.00-3.30) | 0.00 (0.00-3.30) | 0.00 (0.00-3.30) | 37.50 (29.80-45.88) | 3.68 (1.35-8.54) | 1.47 (0.07-5.54) | |  |  |  |
| 51. Personal and protective services workers | 544 | 1.02 (0.94-1.11) | 1.10 (0.45-2.45) | 3.13 (1.92-4.98) | 0.00 (0.00-0.85) | 0.00 (0.00-0.85) | 0.00 (0.00-0.85) | 34.01 (30.15-38.09) | 2.02 (1.09-3.63) | 1.47 (0.69-2.93) | |  |  |  |
| 73. Precision, handicraft, craft printing and related trade workers | 150 | 1.03 (0.88-1.21) | 1.33 (0.06-5.04) | 2.67 (0.81-6.89) | 0.00 (0.00-3.00) | 0.67 (0.00-4.06) | 0.00 (0.00-3.00) | 36.67 (29.37-44.63) | 4.00 (1.66-8.64) | 1.33 (0.06-5.04) | |  |  |  |
| 93. Labourers in mining, construction, manufacturing and transport | 860 | 1.06 (0.99-1.13) | 1.74 (1.03-2.88) | 1.51 (0.86-2.60) | 0.00 (0.00-0.54) | 0.23 (0.01-0.90) | 0.12 (0.00-0.73) | 32.21 (29.17-35.41) | 1.40 (0.77-2.45) | 2.21 (1.40-3.45) | |  |  |  |
| 41. Office clerks | 677 | 1.08 (1.00-1.17) | 1.03 (0.46-2.16) | 2.36 (1.43-3.83) | 0.00 (0.00-0.68) | 0.00 (0.00-0.68) | 0.00 (0.00-0.68) | 32.05 (28.65-35.66) | 1.33 (0.66-2.55) | 3.10 (2.01-4.72) | |  |  |  |
| 83. Drivers and mobile plant operators | 514 | 1.11 (1.02-1.21) | 0.19 (0.00-1.21) | 1.75 (0.87-3.35) | 0.00 (0.00-0.89) | 0.00 (0.00-0.89) | 0.00 (0.00-0.89) | 32.68 (28.77-36.86) | 1.36 (0.60-2.84) | 2.92 (1.74-4.80) | |  |  |  |
| 32. Life science and health associate professionals | 108 | 1.12 (0.93-1.36) | 0.00 (0.00-4.13) | 4.63 (1.72-10.65) | 0.00 (0.00-4.13) | 0.00 (0.00-4.13) | 0.00 (0.00-4.13) | 37.04 (28.51-46.45) | 2.78 (0.60-8.20) | 3.70 (1.14-9.44) | |  |  |  |
| 61. Skilled agricultural and fishery workers | 575 | 1.13 (1.04-1.22) | 0.35 (0.01-1.35) | 1.91 (1.03-3.44) | 0.00 (0.00-0.80) | 0.17 (0.00-1.08) | 0.00 (0.00-0.80) | 40.70 (36.75-44.76) | 1.57 (0.78-3.00) | 2.61 (1.55-4.30) | |  |  |  |
| 81. Stationary plant and related operators | 59 | 1.23 (0.96-1.59) | 0.00 (0.00-7.31) | 1.69 (0.00-9.85) | 0.00 (0.00-7.31) | 0.00 (0.00-7.31) | 0.00 (0.00-7.31) | 42.37 (30.60-55.07) | 1.69 (0.00-9.85) | 3.39 (0.26-12.22) | |  |  |  |
| 92. Agricultural,fishery and related labourers | 35 | 1.42 (1.02-1.98) | 0.00 (0.00-6.88) | 0.00 (0.00-6.88) | 0.00 (0.00-6.88) | 0.00 (0.00-6.88) | 0.00 (0.00-6.88) | 48.57 (32.68-64.69) | 2.86 (0.31-12.59) | 2.86 (0.31-12.59) | |  |  |  |
| Chi2-test for heterogeneity by type of disease P-value with Holm -Bonferroni correction |  |  | 1.12 | 0.00 | 1.86 | 1.69 | 4.01 | 0.00 | 3.67 | 3.63 | |  |  |  |

| Occupation 2-digit ISCO (88) | Nb of suicide | SMR | | | Diseases of the respiratory system | | Diseases of the digestive system | | Diseases of the genitourinary system | | Diseases of the skin and subcutaneous tissues | | Diseases of the musculoskeletal system and connective tissue | | Congenital malformations, deformations and chromosomal abnormalitities | | Accidents, poisoning, traumas (external causes) | | Endocrine, nutritional and metabolic diseases | | |  |  |
| --- | --- | --- | --- | --- | --- | --- | --- | --- | --- | --- | --- | --- | --- | --- | --- | --- | --- | --- | --- | --- | --- | --- | --- |
| 11. Legislators and senior officials | 36 | 0.55 (0.39-0.76) | | | 0.00 (0.00-6.69) | | 0.00 (0.00-6.69) | | 0.00 (0.00-6.69) | | 0.00 (0.00-6.69) | | 0.00 (0.00-6.69) | | 0.00 (0.00-6.69) | | 25.00 (13.15-40.69) | | | 0.00 (0.00-6.69) | | |  |
| 23. Teaching professionals | 165 | 0.65 (0.56-0.76) | | | 0.61 (0.00-3.70) | | 1.21 (0.05-4.59) | | 0.61 (0.00-3.70) | | 0.00 (0.00-2.74) | | 0.00 (0.00-2.74) | | 0.00 (0.00-2.74) | | 18.18 (13.00-24.81) | | | 0.61 (0.00-3.70) | | |  |
| 21. Physical, mathematical and engineering science professionals | 497 | 0.76 (0.69-0.83) | | | 0.60 (0.12-1.85) | | 1.01 (0.36-2.40) | | 0.40 (0.01-1.55) | | 0.00 (0.00-0.92) | | 0.00 (0.00-0.92) | | 0.20 (0.00-1.25) | | 15.09 (12.20-18.52) | | | 1.41 (0.62-2.94) | | |  |
| 12. Corporate managers | 775 | 0.76 (0.71-0.82) | | | 0.39 (0.08-1.19) | | 0.77 (0.31-1.72) | | 0.13 (0.00-0.80) | | 0.00 (0.00-0.59) | | 0.26 (0.01-1.00) | | 0.00 (0.00-0.59) | | 23.35 (20.51-26.46) | | | 1.16 (0.58-2.23) | | |  |
| 31. Physical and engineering science associate professionals | 495 | 0.78 (0.71-0.85) | | | 0.20 (0.00-1.26) | | 0.61 (0.12-1.85) | | 0.00 (0.00-0.93) | | 0.00 (0.00-0.93) | | 0.40 (0.01-1.56) | | 0.20 (0.00-1.26) | | 17.58 (14.47-21.18) | | | 0.81 (0.24-2.14) | | |  |
| 33. Teaching associate professionals | 121 | 0.84 (0.70-1.00) | | | 0.83 (0.00-4.99) | | 0.83 (0.00-4.99) | | 0.00 (0.00-3.70) | | 0.00 (0.00-3.70) | | 0.00 (0.00-3.70) | | 0.00 (0.00-3.70) | | 17.36 (11.57-25.15) | | | 2.48 (0.53-7.35) | | |  |
| 1. Soldiers | 12 | 0.85 (0.44-1.48) | | | 0.00 (0.00-18.53) | | 0.00 (0.00-18.53) | | 0.00 (0.00-18.53) | | 0.00 (0.00-18.53) | | 0.00 (0.00-18.53) | | 0.00 (0.00-18.53) | | 8.33 (0.91-32.85) | | | 0.00 (0.00-18.53) | | |  |
| 13. Managers of small enterprises | 158 | 0.85 (0.72-0.99) | | | 0.63 (0.00-3.86) | | 0.63 (0.00-3.86) | | 1.27 (0.05-4.79) | | 0.00 (0.00-2.86) | | 1.27 (0.05-4.79) | | 0.00 (0.00-2.86) | | 0.00 (0.00-2.86) | | | 1.27 (0.05-4.79) | | |  |
| 24. Other professionals | 391 | 0.86 (0.78-0.95) | | | 1.28 (0.46-3.05) | | 0.51 (0.01-1.97) | | 0.26 (0.00-1.58) | | 0.00 (0.00-1.17) | | 0.26 (0.00-1.58) | | 0.26 (0.00-1.58) | | 14.83 (11.64-18.71) | | | 1.02 (0.30-2.70) | | |  |
| 80. Plant and machine operators and assemblers | 31 | 0.88 (0.62-1.26) | | | 0.00 (0.00-7.72) | | 0.00 (0.00-7.72) | | 0.00 (0.00-7.72) | | 0.00 (0.00-7.72) | | 3.23 (0.35-14.10) | | 0.00 (0.00-7.72) | | 6.45 (1.36-19.12) | | | 0.00 (0.00-7.72) | | |  |
| 91. Sales and services elementary occupations | 158 | 0.89 (0.76-1.04) | | | 0.63 (0.00-3.86) | | 1.27 (0.05-4.79) | | 0.00 (0.00-2.86) | | 0.00 (0.00-2.86) | | 0.63 (0.00-3.86) | | 0.00 (0.00-2.86) | | 20.89 (15.24-27.91) | | | 1.27 (0.05-4.79) | | |  |
| 52. Models, salespersons and demonstrators | 219 | 0.91 (0.80-1.04) | | | 0.46 (0.00-2.80) | | 0.91 (0.03-3.49) | | 0.00 (0.00-2.08) | | 0.46 (0.00-2.80) | | 0.00 (0.00-2.08) | | 0.00 (0.00-2.08) | | 20.09 (15.30-25.92) | | | 1.37 (0.28-4.14) | | |  |
| 10. Legislators, senior officials and managers | 90 | 0.95 (0.77-1.17) | | | 1.11 (0.00-6.63) | | 2.22 (0.13-8.23) | | 0.00 (0.00-4.91) | | 0.00 (0.00-4.91) | | 1.11 (0.00-6.63) | | 0.00 (0.00-4.91) | | 6.67 (2.82-14.07) | | | 1.11 (0.00-6.63) | | |  |
| 22. Life science and health professionals | 138 | 0.95 (0.81-1.13) | | | 0.72 (0.00-4.40) | | 0.00 (0.00-3.26) | | 0.00 (0.00-3.26) | | 0.00 (0.00-3.26) | | 0.00 (0.00-3.26) | | 0.00 (0.00-3.26) | | 23.19 (16.89-30.93) | | | 1.45 (0.07-5.46) | | |  |
| 72. Metal, machinery and related trades workers | 944 | 0.96 (0.90-1.02) | | | 0.64 (0.26-1.42) | | 0.21 (0.01-0.82) | | 0.11 (0.00-0.66) | | 0.11 (0.00-0.66) | | 0.42 (0.12-1.13) | | 0.11 (0.00-0.66) | | 22.46 (19.91-25.23) | | | 0.64 (0.26-1.42) | | |  |
| 71. Extraction and building trades workers | 1022 | 0.96 (0.91-1.02) | | | 0.68 (0.30-1.44) | | 0.49 (0.17-1.18) | | 0.20 (0.00-0.76) | | 0.10 (0.00-0.61) | | 0.78 (0.37-1.57) | | 0.00 (0.00-0.45) | | 21.04 (18.65-23.64) | | | 1.57 (0.95-2.55) | | |  |
| 82. Machine operators and assemblers | 262 | 0.97 (0.86-1.09) | | | 0.38 (0.00-2.35) | | 0.38 (0.00-2.35) | | 0.00 (0.00-1.74) | | 0.38 (0.00-2.35) | | 1.91 (0.69-4.52) | | 0.00 (0.00-1.74) | | 24.81 (19.96-30.39) | | | 1.53 (0.45-4.00) | | |  |
| 34. Other associate professionals | 1012 | 1.00 (0.94-1.07) | | | 0.79 (0.37-1.58) | | 0.40 (0.11-1.05) | | 0.10 (0.00-0.62) | | 0.10 (0.00-0.62) | | 0.59 (0.24-1.32) | | 0.00 (0.00-0.46) | | 23.42 (20.91-26.13) | | | 0.99 (0.51-1.84) | | |  |
| 74. Other craft and related trades workers | 380 | 1.01 (0.91-1.11) | | | 0.26 (0.00-1.63) | | 1.05 (0.31-2.78) | | 0.00 (0.00-1.21) | | 0.26 (0.00-1.63) | | 1.58 (0.64-3.49) | | 0.00 (0.00-1.21) | | 22.11 (18.21-26.55) | | | 0.26 (0.00-1.63) | | |  |
| 42. Customer services clerks | 136 | 1.02 (0.86-1.20) | | | 0.74 (0.00-4.46) | | 0.00 (0.00-3.30) | | 0.00 (0.00-3.30) | | 0.00 (0.00-3.30) | | 0.00 (0.00-3.30) | | 0.00 (0.00-3.30) | | 19.12 (13.34-26.59) | | | 0.74 (0.00-4.46) | | |  |
| 51. Personal and protective services workers | 544 | 1.02 (0.94-1.11) | | | 0.37 (0.01-1.42) | | 0.74 (0.21-1.95) | | 0.00 (0.00-0.85) | | 0.00 (0.00-0.85) | | 0.74 (0.21-1.95) | | 0.00 (0.00-0.85) | | 19.49 (16.37-23.03) | | | 1.47 (0.69-2.93) | | |  |
| 73. Precision, handicraft, craft printing and related trade workers | 150 | 1.03 (0.88-1.21) | | | 0.67 (0.00-4.06) | | 0.00 (0.00-3.00) | | 0.67 (0.00-4.06) | | 0.00 (0.00-3.00) | | 0.00 (0.00-3.00) | | 0.67 (0.00-4.06) | | 18.67 (13.19-25.70) | | | 0.67 (0.00-4.06) | | |  |
| 93. Labourers in mining, construction, manufacturing and transport | 860 | 1.06 (0.99-1.13) | | | 0.58 (0.21-1.40) | | 0.58 (0.21-1.40) | | 0.23 (0.01-0.90) | | 0.12 (0.00-0.73) | | 0.23 (0.01-0.90) | | 0.12 (0.00-0.73) | | 39.77 (36.55-43.08) | | | 1.28 (0.69-2.31) | | |  |
| 41. Office clerks | 677 | 1.08 (1.00-1.17) | | | 0.59 (0.17-1.57) | | 0.74 (0.26-1.77) | | 0.30 (0.01-1.14) | | 0.30 (0.01-1.14) | | 0.59 (0.17-1.57) | | 0.00 (0.00-0.68) | | 22.45 (19.47-25.75) | | | 0.89 (0.36-1.97) | | |  |
| 83. Drivers and mobile plant operators | 514 | 1.11 (1.02-1.21) | | | 0.39 (0.01-1.50) | | 0.97 (0.35-2.33) | | 0.00 (0.00-0.89) | | 0.19 (0.00-1.21) | | 0.58 (0.11-1.79) | | 0.19 (0.00-1.21) | | 20.82 (17.52-24.54) | | | 1.95 (1.01-3.59) | | |  |
| 32. Life science and health associate professionals | 108 | 1.12 (0.93-1.36) | | | 1.85 (0.10-6.91) | | 0.00 (0.00-4.13) | | 0.00 (0.00-4.13) | | 0.00 (0.00-4.13) | | 0.00 (0.00-4.13) | | 0.00 (0.00-4.13) | | 13.89 (8.49-21.77) | | | 2.78 (0.60-8.20) | | |  |
| 61. Skilled agricultural and fishery workers | 575 | 1.13 (1.04-1.22) | | | 1.22 (0.54-2.54) | | 0.70 (0.20-1.84) | | 0.17 (0.00-1.08) | | 0.17 (0.00-1.08) | | 2.09 (1.16-3.65) | | 0.17 (0.00-1.08) | | 20.35 (17.25-23.84) | | | 1.91 (1.03-3.44) | | |  |
| 81. Stationary plant and related operators | 59 | 1.23 (0.96-1.59) | | | 1.69 (0.00-9.85) | | 0.00 (0.00-7.31) | | 1.69 (0.00-9.85) | | 0.00 (0.00-7.31) | | 0.00 (0.00-7.31) | | 0.00 (0.00-7.31) | | 15.25 (8.01-26.75) | | | 0.00 (0.00-7.31) | | |  |
| 92. Agricultural,fishery and related labourers | 35 | 1.42 (1.02-1.98) | | | 0.00 (0.00-6.88) | | 0.00 (0.00-6.88) | | 0.00 (0.00-6.88) | | 0.00 (0.00-6.88) | | 0.00 (0.00-6.88) | | 0.00 (0.00-6.88) | | 25.71 (13.55-41.71) | | | 0.00 (0.00-6.88) | | |  |
| Chi2-test for heterogeneity by type of disease  P-value with Holm-Bonferroni correction |  |  | | | 1.97 | | 3.78 | | 3.27 | | 1.00 | | 0.04 | | 2.90 | | 0.00 | | | 4.37 | | |  |
|  | | |  |  | |  | |  | |  | |  | |  | |  | |  | | |  | | |

| Occupation 2-digit ISCO (88) | Nb of suicide | | SMR | | Substance-related and addictive disorders | | Schizophrenia spectrum and other psychotic disorders | | Mood disorders | | Anxiety disorders/obsessive-compulsive and related disorders | | Personality disorders/gender dysphoria/disruptive, impulse-control and conduct disorders/paraphilic disorders | |  |
| --- | --- | --- | --- | --- | --- | --- | --- | --- | --- | --- | --- | --- | --- | --- | --- |
| 11. Legislators and senior officials | | 36 | | 0.55 (0.39-0.76) | | 0.00 (0.00-6.69) | | 2.78 (0.30-12.26) | | 11.11 (3.87-24.29) | | 0.00 (0.00-6.69) | | 0.00 (0.00-6.69) | |
| 23. Teaching professionals | | 165 | | 0.65 (0.56-0.76) | | 3.03 (1.11-7.09) | | 8.48 (5.02-13.84) | | 25.45 (19.40-32.63) | | 0.00 (0.00-2.74) | | 0.00 (0.00-2.74) | |
| 21. Physical, mathematical and engineering science professionals | | 497 | | 0.76 (0.69-0.83) | | 6.04 (4.23-8.51) | | 5.03 (3.40-7.35) | | 22.13 (18.70-25.99) | | 0.00 (0.00-0.92) | | 0.20 (0.00-1.25) | |
| 12. Corporate managers | | 775 | | 0.76 (0.71-0.82) | | 6.19 (4.69-8.13) | | 3.74 (2.60-5.34) | | 18.97 (16.36-21.88) | | 0.00 (0.00-0.59) | | 0.13 (0.00-0.80) | |
| 31. Physical and engineering science associate professionals | | 495 | | 0.78 (0.71-0.85) | | 8.08 (5.97-10.84) | | 4.44 (2.92-6.67) | | 21.41 (18.02-25.25) | | 0.00 (0.00-0.93) | | 0.61 (0.12-1.85) | |
| 33. Teaching associate professionals | | 121 | | 0.84 (0.70-1.00) | | 7.44 (3.79-13.70) | | 4.13 (1.53-9.56) | | 30.58 (23.05-39.30) | | 0.83 (0.00-4.99) | | 0.00 (0.00-3.70) | |
| 1. Soldiers | | 12 | | 0.85 (0.44-1.48) | | 0.00 (0.00-18.53) | | 0.00 (0.00-18.53) | | 33.33 (12.45-61.24) | | 0.00 (0.00-18.53) | | 8.33 (0.91-32.85) | |
| 13. Managers of small enterprises | | 158 | | 0.85 (0.72-0.99) | | 6.96 (3.81-12.16) | | 1.90 (0.40-5.69) | | 23.42 (17.46-30.63) | | 0.00 (0.00-2.86) | | 0.00 (0.00-2.86) | |
| 24. Other professionals | | 391 | | 0.86 (0.78-0.95) | | 9.46 (6.92-12.80) | | 5.88 (3.91-8.71) | | 23.79 (19.83-28.26) | | 0.77 (0.15-2.34) | | 0.51 (0.01-1.97) | |
| 80. Plant and machine operators and assemblers | | 31 | | 0.88 (0.62-1.26) | | 9.68 (2.80-23.63) | | 0.00 (0.00-7.72) | | 32.26 (17.94-49.71) | | 0.00 (0.00-7.72) | | 0.00 (0.00-7.72) | |
| 91. Sales and services elementary occupations | | 158 | | 0.89 (0.76-1.04) | | 7.59 (4.28-12.92) | | 5.70 (2.88-10.61) | | 19.62 (14.14-26.54) | | 3.16 (1.16-7.39) | | 0.63 (0.00-3.86) | |
| 52. Models, salespersons and demonstrators | | 219 | | 0.91 (0.80-1.04) | | 7.76 (4.83-12.15) | | 2.28 (0.83-5.38) | | 22.37 (17.34-28.36) | | 0.46 (0.00-2.80) | | 0.00 (0.00-2.08) | |
| 10. Legislators, senior officials and managers | | 90 | | 0.95 (0.77-1.17) | | 4.44 (1.39-11.23) | | 0.00 (0.00-4.91) | | 28.89 (20.50-39.00) | | 0.00 (0.00-4.91) | | 0.00 (0.00-4.91) | |
| 22. Life science and health professionals | | 138 | | 0.95 (0.81-1.13) | | 4.35 (1.81-9.36) | | 2.90 (0.88-7.47) | | 17.39 (11.91-24.63) | | 1.45 (0.07-5.46) | | 0.00 (0.00-3.26) | |
| 72. Metal, machinery and related trades workers | | 944 | | 0.96 (0.90-1.02) | | 9.22 (7.53-11.24) | | 6.99 (5.52-8.81) | | 21.19 (18.70-23.91) | | 0.95 (0.47-1.83) | | 0.53 (0.19-1.27) | |
| 71. Extraction and building trades workers | | 1022 | | 0.96 (0.91-1.02) | | 11.06 (9.27-13.13) | | 4.79 (3.63-6.29) | | 19.86 (17.53-22.42) | | 0.59 (0.24-1.31) | | 0.39 (0.11-1.04) | |
| 82. Machine operators and assemblers | | 262 | | 0.97 (0.86-1.09) | | 9.92 (6.82-14.19) | | 5.34 (3.14-8.84) | | 23.28 (18.56-28.78) | | 1.15 (0.23-3.47) | | 0.00 (0.00-1.74) | |
| 34. Other associate professionals | | 1012 | | 1.00 (0.94-1.07) | | 6.42 (5.06-8.11) | | 4.05 (2.99-5.46) | | 21.25 (18.83-23.87) | | 0.40 (0.11-1.05) | | 0.40 (0.11-1.05) | |
| 74. Other craft and related trades workers | | 380 | | 1.01 (0.91-1.11) | | 11.58 (8.72-15.21) | | 4.74 (2.97-7.41) | | 18.16 (14.59-22.36) | | 0.53 (0.02-2.03) | | 0.26 (0.00-1.63) | |
| 42. Customer services clerks | | 136 | | 1.02 (0.86-1.20) | | 6.62 (3.36-12.26) | | 7.35 (3.90-13.15) | | 25.74 (19.10-33.71) | | 0.00 (0.00-3.30) | | 0.74 (0.00-4.46) | |
| 51. Personal and protective services workers | | 544 | | 1.02 (0.94-1.11) | | 9.19 (7.02-11.93) | | 5.88 (4.17-8.21) | | 20.77 (17.57-24.39) | | 0.37 (0.01-1.42) | | 0.00 (0.00-0.85) | |
| 73. Precision, handicraft, craft printing and related trade workers | | 150 | | 1.03 (0.88-1.21) | | 6.67 (3.52-11.97) | | 3.33 (1.22-7.77) | | 26.00 (19.62-33.58) | | 1.33 (0.06-5.04) | | 0.67 (0.00-4.06) | |
| 93. Labourers in mining, construction, manufacturing and transport | | 860 | | 1.06 (0.99-1.13) | | 11.40 (9.43-13.70) | | 9.30 (7.53-11.44) | | 13.72 (11.58-16.19) | | 0.35 (0.07-1.07) | | 0.35 (0.07-1.07) | |
| 41. Office clerks | | 677 | | 1.08 (1.00-1.17) | | 8.71 (6.80-11.09) | | 6.06 (4.48-8.13) | | 19.05 (16.27-22.19) | | 0.44 (0.09-1.36) | | 0.30 (0.01-1.14) | |
| 83. Drivers and mobile plant operators | | 514 | | 1.11 (1.02-1.21) | | 9.34 (7.10-12.18) | | 4.86 (3.29-7.11) | | 20.04 (16.80-23.72) | | 0.19 (0.00-1.21) | | 0.58 (0.11-1.79) | |
| 32. Life science and health associate professionals | | 108 | | 1.12 (0.93-1.36) | | 12.04 (7.04-19.64) | | 3.70 (1.14-9.44) | | 24.07 (16.94-32.99) | | 0.00 (0.00-4.13) | | 0.93 (0.00-5.57) | |
| 61. Skilled agricultural and fishery workers | | 575 | | 1.13 (1.04-1.22) | | 6.96 (5.13-9.35) | | 6.61 (4.83-8.96) | | 28.17 (24.65-31.99) | | 0.52 (0.10-1.60) | | 0.70 (0.20-1.84) | |
| 81. Stationary plant and related operators | | 59 | | 1.23 (0.96-1.59) | | 8.47 (3.27-18.75) | | 6.78 (2.20-16.64) | | 25.42 (15.96-37.89) | | 0.00 (0.00-7.31) | | 0.00 (0.00-7.31) | |
| 92. Agricultural,fishery and related labourers | | 35 | | 1.42 (1.02-1.98) | | 22.86 (11.44-38.55) | | 8.57 (2.47-21.14) | | 25.71 (13.55-41.71) | | 0.00 (0.00-6.88) | | 0.00 (0.00-6.88) | |
|  | |  | |  | |  | |  | |  | |  | |  | |
| Chi2-test for heterogeneity by type of disease  P-value with Holm-Bonferroni correction | |  | |  | | 0.00 | | 0.00 | | 0.00 | | 0.02 | | 0.39 | |

^a^Based on the International Standard Classification of Occupations (2-digit ISCO 88)

^b^ Only categories with at least 10 suicides are presented.

^c^ Standardized Mortality Ratio for suicide
