# Supplementary material for: Prevalence of somatic and psychiatric morbidity across occupations in Switzerland and its correlation with suicide mortality: results from the Swiss National Cohort (1990–2014)
Source: BMC Psychiatry. 2020 Jun 22;20:324. doi: 10.1186/s12888-020-02733-7 (PMC7310107; doi:10.1186/s12888-020-02733-7)
Supplement: Supplementary file 2 — Additional file 2 Table S2. Pearson’s Chi2 test of heterogeneity in prevalence of reported diseases with associated 95% confidence interval across occupational groupsa among female workers deceased by suicide (SNC, 1990–2014) [file 12888_2020_2733_MOESM2_ESM.docx]

| **Table S2.** Pearson’s Chi2 test of heterogeneity in prevalence of reported diseases with associated 95% confidence interval across occupational groups^a^ among female workers deceased by suicide (SNC, 1990-2014) | | | | | | | | | | |  |  |  |  |
| --- | --- | --- | --- | --- | --- | --- | --- | --- | --- | --- | --- | --- | --- | --- |
| Occupation 2-digit ISCO (88) | Nb of suicide^b^ | SMR^c^ | Infectious and  parasitic diseases | Malignant neoplasms | Benign neoplasms | Neoplasms of uncertain or unknown behaviour | Mental and behavioural disorders | Diseases of the nervous system and sense organs | Diseases of the circulatory system |  |  |  |  |  |
| 74. Other craft and related trades workers | 29 | 0.65 (0.44-0.93) | 0.00 (0.00-8.23) | 6.90 (1.46-20.33) | 0.00 (0.00-8.23) | 0.00 (0.00-8.23) | 34.48 (19.30-52.59) | 3.45 (0.37-15.01) | 0.00 (0.00-8.23) |  |  |  |  |  |
| 61. Skilled agricultural and fishery workers | 46 | 0.65 (0.49-0.87) | 0.00 (0.00-9.20) | 4.35 (0.39-15.34) | 0.00 (0.00-9.20) | 0.00 (0.00-9.20) | 45.65 (32.15-59.82) | 2.17 (0.00-12.38) | 2.17 (0.00-12.38) |  |  |  |  |  |
| 13. Managers of small enterprises | 23 | 0.68 (0.43-1.02) | 0.00 (0.00-10.24) | 4.35 (0.47-18.58) | 0.00 (0.00-10.24) | 0.00 (0.00-10.24) | 43.48 (24.99-63.50) | 4.35 (0.47-18.58) | 0.00 (0.00-10.24) |  |  |  |  |  |
| 72. Metal, machinery and related trades workers | 12 | 0.69 (0.35-1.20) | 0.00 (0.00-18.53) | 8.33 (0.91-32.85) | 0.00 (0.00-18.53) | 0.00 (0.00-18.53) | 41.67 (18.05-68.81) | 8.33 (0.91-32.85) | 8.33 (0.91-32.85) |  |  |  |  |  |
| 23. Teaching professionals | 78 | 0.78 (0.63-0.98) | 1.28 (0.00-7.59) | 11.54 (5.98-20.71) | 1.28 (0.00-7.59) | 0.00 (0.00-5.63) | 43.59 (33.14-54.64) | 2.56 (0.16-9.42) | 2.56 (0.16-9.42) |  |  |  |  |  |
| 12. Corporate managers | 87 | 0.84 (0.68-1.04) | 0.00 (0.00-5.07) | 11.49 (6.18-20.07) | 0.00 (0.00-5.07) | 0.00 (0.00-5.07) | 44.83 (34.81-55.28) | 2.30 (0.14-8.49) | 0.00 (0.00-5.07) |  |  |  |  |  |
| 91. Sales and services elementary occupations | 113 | 0.84 (0.69-1.00) | 0.88 (0.00-5.33) | 6.19 (2.82-12.45) | 0.00 (0.00-3.95) | 0.00 (0.00-3.95) | 42.48 (33.76-51.69) | 3.54 (1.09-9.04) | 0.00 (0.00-3.95) |  |  |  |  |  |
| 52. Models, salespersons and demonstrators | 216 | 0.87 (0.76-0.99) | 0.00 (0.00-2.10) | 4.63 (2.43-8.42) | 0.00 (0.00-2.10) | 0.00 (0.00-2.10) | 48.15 (41.58-54.79) | 4.17 (2.10-7.84) | 0.46 (0.00-2.84) |  |  |  |  |  |
| 33. Teaching associate professionals | 108 | 0.88 (0.73-1.06) | 0.93 (0.00-5.57) | 10.19 (5.63-17.48) | 0.00 (0.00-4.13) | 0.00 (0.00-4.13) | 37.04 (28.51-46.45) | 3.70 (1.14-9.44) | 0.00 (0.00-4.13) |  |  |  |  |  |
| 31. Physical and engineering science associate professionals | 55 | 0.93 (0.71-1.21) | 1.82 (0.00-10.51) | 12.73 (6.00-24.32) | 0.00 (0.00-7.80) | 0.00 (0.00-7.80) | 43.64 (31.37-56.74) | 5.45 (1.29-15.44) | 0.00 (0.00-7.80) |  |  |  |  |  |
| 41. Office clerks | 515 | 0.93 (0.85-1.01) | 0.39 (0.01-1.50) | 7.38 (5.40-9.99) | 0.19 (0.00-1.21) | 0.00 (0.00-0.89) | 43.11 (38.90-47.42) | 2.14 (1.15-3.83) | 1.17 (0.47-2.58) |  |  |  |  |  |
| 82. Machine operators and assemblers | 31 | 0.97 (0.68-1.38) | 0.00 (0.00-7.72) | 6.45 (1.36-19.12) | 0.00 (0.00-7.72) | 0.00 (0.00-7.72) | 38.71 (23.19-56.23) | 3.23 (0.35-14.10) | 3.23 (0.35-14.10) |  |  |  |  |  |
| 10. Legislators, senior officials and managers | 16 | 0.99 (0.57-1.62) | 0.00 (0.00-14.32) | 12.50 (2.69-34.42) | 0.00 (0.00-14.32) | 6.25 (0.68-25.69) | 43.75 (22.16-67.39) | 6.25 (0.68-25.69) | 6.25 (0.68-25.69) |  |  |  |  |  |
| 32. Life science and health associate professionals | 232 | 1.02 (0.90-1.16) | 0.86 (0.03-3.29) | 5.60 (3.22-9.43) | 0.00 (0.00-1.96) | 0.00 (0.00-1.96) | 48.28 (41.93-54.68) | 2.16 (0.78-5.09) | 2.16 (0.78-5.09) |  |  |  |  |  |
| 51. Personal and protective services workers | 349 | 1.03 (0.93-1.15) | 0.00 (0.00-1.31) | 8.02 (5.57-11.39) | 0.00 (0.00-1.31) | 0.00 (0.00-1.31) | 46.42 (41.25-51.66) | 2.01 (0.89-4.17) | 0.57 (0.02-2.20) |  |  |  |  |  |
| 34. Other associate professionals | 230 | 1.04 (0.91-1.18) | 1.30 (0.26-3.94) | 11.30 (7.78-16.10) | 0.00 (0.00-1.98) | 0.00 (0.00-1.98) | 46.09 (39.76-52.54) | 3.04 (1.36-6.27) | 1.74 (0.52-4.55) |  |  |  |  |  |
| 21. Physical, mathematical and engineering science professionals | 24 | 1.05 (0.67-1.57) | 0.00 (0.00-9.84) | 0.00 (0.00-9.84) | 0.00 (0.00-9.84) | 0.00 (0.00-9.84) | 33.33 (17.20-53.23) | 0.00 (0.00-9.84) | 0.00 (0.00-9.84) |  |  |  |  |  |
| 42. Customer services clerks | 103 | 1.10 (0.91-1.33) | 0.97 (0.00-5.83) | 6.80 (3.10-13.59) | 0.00 (0.00-4.32) | 0.00 (0.00-4.32) | 42.72 (33.59-52.37) | 2.91 (0.63-8.58) | 1.94 (0.10-7.23) |  |  |  |  |  |
| 73. Precision, handicraft, craft printing and related trade workers | 31 | 1.15 (0.81-1.63) | 0.00 (0.00-7.72) | 9.68 (2.80-23.63) | 0.00 (0.00-7.72) | 0.00 (0.00-7.72) | 35.48 (20.53-53.01) | 6.45 (1.36-19.12) | 0.00 (0.00-7.72) |  |  |  |  |  |
| 93. Labourers in mining, construction, manufacturing and transport | 263 | 1.19 (1.05-1.34) | 2.28 (0.93-5.00) | 2.66 (1.19-5.50) | 0.00 (0.00-1.73) | 0.00 (0.00-1.73) | 38.78 (33.09-44.80) | 1.52 (0.45-3.99) | 0.76 (0.03-2.91) |  |  |  |  |  |
| 22. Life science and health professionals | 31 | 1.22 (0.86-1.74) | 0.00 (0.00-7.72) | 12.90 (4.52-27.82) | 0.00 (0.00-7.72) | 0.00 (0.00-7.72) | 35.48 (20.53-53.01) | 3.23 (0.35-14.10) | 0.00 (0.00-7.72) |  |  |  |  |  |
| 24. Other professionals | 133 | 1.22 (1.03-1.44) | 0.00 (0.00-3.37) | 12.78 (8.04-19.61) | 0.75 (0.00-4.56) | 0.75 (0.00-4.56) | 29.32 (22.23-37.57) | 4.51 (1.88-9.70) | 0.75 (0.00-4.56) |  |  |  |  |  |
| 83. Drivers and mobile plant operators | 13 | 1.83 (0.98-3.14) | 0.00 (0.00-17.26) | 7.69 (0.84-30.71) | 0.00 (0.00-17.26) | 0.00 (0.00-17.26) | 38.46 (16.47-65.00) | 0.00 (0.00-17.26) | 0.00 (0.00-17.26) |  |  |  |  |  |
| Chi2-test for heterogeneity by type of disease  P-value with Holm-Bonferroni correction |  |  | 4.45 | 0.95 | 4.59 | 0.00 | 1.58 | 0.00 | 3.63 |  |  |  |  |  |

| Occupation 2-digit ISCO (88) | Nb of suicide | SMR | | | Diseases of the respiratory system | | Diseases of the digestive system | | Diseases of the genitourinary system | | Diseases of the skin and subcutaneous tissues | | Diseases of the musculoskeletal system and connective tissue | | Congenital malformations, deformations and chromosomal abnormalitities | | Accidents, poisoning, traumas (external causes) | | Endocrine, nutritional and metabolic diseases | | |  |  |
| --- | --- | --- | --- | --- | --- | --- | --- | --- | --- | --- | --- | --- | --- | --- | --- | --- | --- | --- | --- | --- | --- | --- | --- |
| 74. Other craft and related trades workers | 29 | 0.65 (0.44-0.93) | | | 0.00 (0.00-8.23) | | 0.00 (0.00-8.23) | | 0.00 (0.00-8.23) | | 0.00 (0.00-8.23) | | 3.45 (0.37-15.01) | | 0.00 (0.00-8.23) | | 13.79 (4.84-29.54) | | 0.00 (0.00-8.23) | | |  |  |
| 61. Skilled agricultural and fishery workers | 46 | 0.65 (0.49-0.87) | | | 0.00 (0.00-9.20) | | 0.00 (0.00-9.20) | | 0.00 (0.00-9.20) | | 0.00 (0.00-9.20) | | 0.00 (0.00-9.20) | | 0.00 (0.00-9.20) | | 13.04 (5.74-26.04) | | 0.00 (0.00-9.20) | | |  |  |
| 13. Managers of small enterprises | 23 | 0.68 (0.43-1.02) | | | 4.35 (0.47-18.58) | | 0.00 (0.00-10.24) | | 4.35 (0.47-18.58) | | 0.00 (0.00-10.24) | | 0.00 (0.00-10.24) | | 0.00 (0.00-10.24) | | 13.04 (3.81-30.87) | | 0.00 (0.00-10.24) | | |  |  |
| 72. Metal, machinery and related trades workers | 12 | 0.69 (0.35-1.20) | | | 0.00 (0.00-18.53) | | 0.00 (0.00-18.53) | | 0.00 (0.00-18.53) | | 0.00 (0.00-18.53) | | 0.00 (0.00-18.53) | | 0.00 (0.00-18.53) | | 16.67 (3.63-43.62) | | 0.00 (0.00-18.53) | | |  |  |
| 23. Teaching professionals | 78 | 0.78 (0.63-0.98) | | | 0.00 (0.00-5.63) | | 1.28 (0.00-7.59) | | 0.00 (0.00-5.63) | | 0.00 (0.00-5.63) | | 1.28 (0.00-7.59) | | 0.00 (0.00-5.63) | | 14.10 (7.89-23.69) | | 1.28 (0.00-7.59) | | |  |  |
| 12. Corporate managers | 87 | 0.84 (0.68-1.04) | | | 1.15 (0.00-6.84) | | 1.15 (0.00-6.84) | | 0.00 (0.00-5.07) | | 0.00 (0.00-5.07) | | 1.15 (0.00-6.84) | | 0.00 (0.00-5.07) | | 25.29 (17.28-35.39) | | 0.00 (0.00-5.07) | | |  |  |
| 91. Sales and services elementary occupations | 113 | 0.84 (0.69-1.00) | | | 0.00 (0.00-3.95) | | 0.00 (0.00-3.95) | | 0.00 (0.00-3.95) | | 0.00 (0.00-3.95) | | 0.88 (0.00-5.33) | | 0.00 (0.00-3.95) | | 20.35 (13.90-28.76) | | 0.00 (0.00-3.95) | | |  |  |
| 52. Models, salespersons and demonstrators | 216 | 0.87 (0.76-0.99) | | | 2.78 (1.14-6.07) | | 0.46 (0.00-2.84) | | 0.00 (0.00-2.10) | | 0.00 (0.00-2.10) | | 0.46 (0.00-2.84) | | 0.00 (0.00-2.10) | | 21.76 (16.75-27.75) | | 0.93 (0.03-3.53) | | |  |  |
| 33. Teaching associate professionals | 108 | 0.88 (0.73-1.06) | | | 0.93 (0.00-5.57) | | 0.00 (0.00-4.13) | | 0.00 (0.00-4.13) | | 0.00 (0.00-4.13) | | 0.93 (0.00-5.57) | | 0.00 (0.00-4.13) | | 20.37 (13.79-28.99) | | 1.85 (0.10-6.91) | | |  |  |
| 31. Physical and engineering science associate professionals | 55 | 0.93 (0.71-1.21) | | | 5.45 (1.29-15.44) | | 0.00 (0.00-7.80) | | 0.00 (0.00-7.80) | | 0.00 (0.00-7.80) | | 0.00 (0.00-7.80) | | 0.00 (0.00-7.80) | | 14.55 (7.29-26.43) | | 0.00 (0.00-7.80) | | |  |  |
| 41. Office clerks | 515 | 0.93 (0.85-1.01) | | | 0.58 (0.11-1.78) | | 0.39 (0.01-1.50) | | 0.00 (0.00-0.89) | | 0.19 (0.00-1.21) | | 0.39 (0.01-1.50) | | 0.00 (0.00-0.89) | | 17.86 (14.79-21.42) | | 0.78 (0.23-2.06) | | |  |  |
| 82. Machine operators and assemblers | 31 | 0.97 (0.68-1.38) | | | 3.23 (0.35-14.10) | | 0.00 (0.00-7.72) | | 0.00 (0.00-7.72) | | 0.00 (0.00-7.72) | | 0.00 (0.00-7.72) | | 3.23 (0.35-14.10) | | 16.13 (6.43-31.80) | | 0.00 (0.00-7.72) | | |  |  |
| 10. Legislators, senior officials and managers | 16 | 0.99 (0.57-1.62) | | | 0.00 (0.00-14.32) | | 0.00 (0.00-14.32) | | 0.00 (0.00-14.32) | | 0.00 (0.00-14.32) | | 6.25 (0.68-25.69) | | 0.00 (0.00-14.32) | | 6.25 (0.68-25.69) | | 0.00 (0.00-14.32) | | |  |  |
| 32. Life science and health associate professionals | 232 | 1.02 (0.90-1.16) | | | 0.43 (0.00-2.65) | | 0.43 (0.00-2.65) | | 0.00 (0.00-1.96) | | 0.00 (0.00-1.96) | | 1.29 (0.26-3.91) | | 0.43 (0.00-2.65) | | 16.38 (12.14-21.71) | | 2.59 (1.06-5.66) | | |  |  |
| 51. Personal and protective services workers | 349 | 1.03 (0.93-1.15) | | | 0.29 (0.00-1.77) | | 0.00 (0.00-1.31) | | 0.00 (0.00-1.31) | | 0.29 (0.00-1.77) | | 0.29 (0.00-1.77) | | 0.00 (0.00-1.31) | | 22.06 (18.02-26.72) | | 1.15 (0.34-3.02) | | |  |  |
| 34. Other associate professionals | 230 | 1.04 (0.91-1.18) | | | 0.87 (0.03-3.32) | | 0.87 (0.03-3.32) | | 0.00 (0.00-1.98) | | 0.00 (0.00-1.98) | | 0.87 (0.03-3.32) | | 0.00 (0.00-1.98) | | 13.04 (9.25-18.05) | | 1.74 (0.52-4.55) | | |  |  |
| 21. Physical, mathematical and engineering science professionals | 24 | 1.05 (0.67-1.57) | | | 0.00 (0.00-9.84) | | 0.00 (0.00-9.84) | | 0.00 (0.00-9.84) | | 0.00 (0.00-9.84) | | 0.00 (0.00-9.84) | | 0.00 (0.00-9.84) | | 12.50 (3.65-29.73) | | 0.00 (0.00-9.84) | | |  |  |
| 42. Customer services clerks | 103 | 1.10 (0.91-1.33) | | | 0.00 (0.00-4.32) | | 0.00 (0.00-4.32) | | 0.00 (0.00-4.32) | | 0.00 (0.00-4.32) | | 2.91 (0.63-8.58) | | 0.00 (0.00-4.32) | | 16.50 (10.47-24.95) | | 1.94 (0.10-7.23) | | |  |  |
| 73. Precision, handicraft, craft printing and related trade workers | 31 | 1.15 (0.81-1.63) | | | 3.23 (0.35-14.10) | | 0.00 (0.00-7.72) | | 0.00 (0.00-7.72) | | 0.00 (0.00-7.72) | | 0.00 (0.00-7.72) | | 0.00 (0.00-7.72) | | 12.90 (4.52-27.82) | | 0.00 (0.00-7.72) | | |  |  |
| 93. Labourers in mining, construction, manufacturing and transport | 263 | 1.19 (1.05-1.34) | | | 0.38 (0.00-2.34) | | 0.38 (0.00-2.34) | | 0.38 (0.00-2.34) | | 0.00 (0.00-1.73) | | 1.52 (0.45-3.99) | | 0.00 (0.00-1.73) | | 35.36 (29.83-41.32) | | 0.76 (0.03-2.91) | | |  |  |
| 22. Life science and health professionals | 31 | 1.22 (0.86-1.74) | | | 0.00 (0.00-7.72) | | 0.00 (0.00-7.72) | | 0.00 (0.00-7.72) | | 0.00 (0.00-7.72) | | 0.00 (0.00-7.72) | | 0.00 (0.00-7.72) | | 32.26 (17.94-49.71) | | 0.00 (0.00-7.72) | | |  |  |
| 24. Other professionals | 133 | 1.22 (1.03-1.44) | | | 0.75 (0.00-4.56) | | 0.75 (0.00-4.56) | | 0.75 (0.00-4.56) | | 0.75 (0.00-4.56) | | 3.01 (0.92-7.74) | | 0.75 (0.00-4.56) | | 18.05 (12.38-25.51) | | 0.75 (0.00-4.56) | | |  |  |
| 83. Drivers and mobile plant operators | 13 | 1.83 (0.98-3.14) | | | 0.00 (0.00-17.26) | | 0.00 (0.00-17.26) | | 0.00 (0.00-17.26) | | 0.00 (0.00-17.26) | | 0.00 (0.00-17.26) | | 0.00 (0.00-17.26) | | 30.77 (11.39-57.70) | | 0.00 (0.00-17.26) | | |  |  |
|  | | |  |  | |  | |  | |  | |  | |  | |  | |  | | |  | | |
| Chi2-test for heterogeneity by type of disease  P-value with Holm-Bonferroni correction |  |  | | | 0.81 | | 2.00 | | 0.12 | | 1.00 | | 0.97 | | 0.94 | | 0.00 | | 2.92 | | |  |  |
|  | | |  |  | |  | |  | |  | |  | |  | |  | |  | |  | | |  |

| Occupation 2-digit ISCO (88) | Nb of suicide | | SMR | | Substance-related and addictive disorders | | Schizophrenia spectrum and other psychotic disorders | | Mood disorders | | Anxiety disorders/obsessive-compulsive and related disorders | | Personality disorders/gender dysphoria/disruptive, impulse-control and conduct disorders/paraphilic disorders | |  |
| --- | --- | --- | --- | --- | --- | --- | --- | --- | --- | --- | --- | --- | --- | --- | --- |
| 74. Other craft and related trades workers | | 29 | | 0.65 (0.44-0.93) | | 0.00 (0.00-8.23) | | 6.90 (1.46-20.33) | | 20.69 (9.13-37.77) | | 0.00 (0.00-8.23) | | 3.45 (0.37-15.01) | |
| 61. Skilled agricultural and fishery workers | | 46 | | 0.65 (0.49-0.87) | | 4.35 (0.39-15.34) | | 4.35 (0.39-15.34) | | 39.13 (26.37-53.57) | | 0.00 (0.00-9.20) | | 0.00 (0.00-9.20) | |
| 13. Managers of small enterprises | | 23 | | 0.68 (0.43-1.02) | | 8.70 (1.85-25.08) | | 0.00 (0.00-10.24) | | 43.48 (24.99-63.50) | | 0.00 (0.00-10.24) | | 0.00 (0.00-10.24) | |
| 72. Metal, machinery and related trades workers | | 12 | | 0.69 (0.35-1.20) | | 8.33 (0.91-32.85) | | 0.00 (0.00-18.53) | | 41.67 (18.05-68.81) | | 0.00 (0.00-18.53) | | 0.00 (0.00-18.53) | |
| 23. Teaching professionals | | 78 | | 0.78 (0.63-0.98) | | 1.28 (0.00-7.59) | | 8.97 (4.15-17.65) | | 33.33 (23.84-44.39) | | 0.00 (0.00-5.63) | | 0.00 (0.00-5.63) | |
| 12. Corporate managers | | 87 | | 0.84 (0.68-1.04) | | 11.49 (6.18-20.07) | | 9.20 (4.51-17.33) | | 25.29 (17.28-35.39) | | 0.00 (0.00-5.07) | | 0.00 (0.00-5.07) | |
| 91. Sales and services elementary occupations | | 113 | | 0.84 (0.69-1.00) | | 2.65 (0.57-7.85) | | 10.62 (6.04-17.79) | | 29.20 (21.59-38.19) | | 0.88 (0.00-5.33) | | 0.88 (0.00-5.33) | |
| 52. Models, salespersons and demonstrators | | 216 | | 0.87 (0.76-0.99) | | 6.02 (3.46-10.11) | | 8.80 (5.64-13.40) | | 35.65 (29.56-42.24) | | 0.46 (0.00-2.84) | | 0.46 (0.00-2.84) | |
| 33. Teaching associate professionals | | 108 | | 0.88 (0.73-1.06) | | 2.78 (0.60-8.20) | | 4.63 (1.72-10.65) | | 29.63 (21.81-38.85) | | 0.00 (0.00-4.13) | | 0.00 (0.00-4.13) | |
| 31. Physical and engineering science associate professionals | | 55 | | 0.93 (0.71-1.21) | | 0.00 (0.00-7.80) | | 3.64 (0.29-13.04) | | 34.55 (23.32-47.79) | | 0.00 (0.00-7.80) | | 7.27 (2.38-17.75) | |
| 41. Office clerks | | 515 | | 0.93 (0.85-1.01) | | 5.63 (3.92-8.00) | | 6.99 (5.07-9.55) | | 29.90 (26.11-34.00) | | 0.58 (0.11-1.78) | | 1.17 (0.47-2.58) | |
| 82. Machine operators and assemblers | | 31 | | 0.97 (0.68-1.38) | | 6.45 (1.36-19.12) | | 9.68 (2.80-23.63) | | 25.81 (13.03-42.87) | | 0.00 (0.00-7.72) | | 0.00 (0.00-7.72) | |
| 10. Legislators, senior officials and managers | | 16 | | 0.99 (0.57-1.62) | | 0.00 (0.00-14.32) | | 0.00 (0.00-14.32) | | 37.50 (17.43-61.66) | | 0.00 (0.00-14.32) | | 0.00 (0.00-14.32) | |
| 32. Life science and health associate professionals | | 232 | | 1.02 (0.90-1.16) | | 5.60 (3.22-9.43) | | 5.17 (2.89-8.91) | | 36.21 (30.29-42.57) | | 1.29 (0.26-3.91) | | 2.59 (1.06-5.66) | |
| 51. Personal and protective services workers | | 349 | | 1.03 (0.93-1.15) | | 4.01 (2.35-6.68) | | 10.60 (7.76-14.30) | | 30.09 (25.51-35.10) | | 0.86 (0.17-2.62) | | 0.86 (0.17-2.62) | |
| 34. Other associate professionals | | 230 | | 1.04 (0.91-1.18) | | 4.78 (2.60-8.45) | | 8.26 (5.29-12.60) | | 30.00 (24.44-36.22) | | 2.17 (0.79-5.13) | | 1.74 (0.52-4.55) | |
| 21. Physical, mathematical and engineering science professionals | | 24 | | 1.05 (0.67-1.57) | | 8.33 (1.77-24.14) | | 0.00 (0.00-9.84) | | 29.17 (14.10-48.92) | | 0.00 (0.00-9.84) | | 0.00 (0.00-9.84) | |
| 42. Customer services clerks | | 103 | | 1.10 (0.91-1.33) | | 8.74 (4.48-15.97) | | 2.91 (0.63-8.58) | | 33.98 (25.54-43.57) | | 0.00 (0.00-4.32) | | 0.00 (0.00-4.32) | |
| 73. Precision, handicraft, craft printing and related trade workers | | 31 | | 1.15 (0.81-1.63) | | 3.23 (0.35-14.10) | | 3.23 (0.35-14.10) | | 29.03 (15.44-46.34) | | 0.00 (0.00-7.72) | | 0.00 (0.00-7.72) | |
| 93. Labourers in mining, construction, manufacturing and transport | | 263 | | 1.19 (1.05-1.34) | | 9.13 (6.16-13.27) | | 9.51 (6.48-13.70) | | 20.15 (15.73-25.43) | | 0.38 (0.00-2.34) | | 0.38 (0.00-2.34) | |
| 22. Life science and health professionals | | 31 | | 1.22 (0.86-1.74) | | 6.45 (1.36-19.12) | | 6.45 (1.36-19.12) | | 16.13 (6.43-31.80) | | 3.23 (0.35-14.10) | | 0.00 (0.00-7.72) | |
| 24. Other professionals | | 133 | | 1.22 (1.03-1.44) | | 1.50 (0.07-5.66) | | 5.26 (2.38-10.66) | | 21.80 (15.59-29.60) | | 0.00 (0.00-3.37) | | 0.75 (0.00-4.56) | |
| 83. Drivers and mobile plant operators | | 13 | | 1.83 (0.98-3.14) | | 23.08 (6.97-49.74) | | 7.69 (0.84-30.71) | | 15.38 (3.34-40.90) | | 0.00 (0.00-17.26) | | 0.00 (0.00-17.26) | |
| Chi2-test for heterogeneity by type of disease  P-value with Holm-Bonferroni correction | |  | | \|  \| 0.90 \| \| --- \| --- \| | | 0.11 | | 0.90 | | 0.07 | | 0.87 | | 0.29 | |

^a^Based on the International Standard Classification of Occupations (2-digit ISCO 88)

^b^ Only categories with at least 10 suicides are presented.

^c^ Standardized Mortality Ratio for suicide
